# ToxBase: A Multidimensional ToxCast Reference Database for High-Throughput Human Exposome Analysis

**DOI:** 10.1021/acs.est.5c18068

**Published:** 2026-06-09

**Authors:** Ryan Nguyen, Griffin Rangel, Xingzhu Liu, Reuben A. Santoso, Amogh Bantwal, Dylan H. Ross, Ryan P. Seguin, Jennifer Liem, Yvonne S. Lin, Brian Pratt, Brendan X. MacLean, Michael J. MacCoss, Libin Xu

**Affiliations:** † Department of Medicinal Chemistry, 7284 University of Washington, Seattle, Washington 98195, United States; ‡ Department of Pharmaceutics, 7284 University of Washington, Seattle Washington 98195, United States; § Department of Genome Sciences, 7284 University of Washington, Seattle, Washington 98195, United States

**Keywords:** exposome, ToxCast, ion mobility-mass spectrometry, collision cross section, MS/MS, suspect screening

## Abstract

High-resolution mass spectrometry (HRMS) is the gold-standard technique for comprehensively profiling chemical exposures in complex human matrices, making it a powerful analytical tool for advancing human exposome research. Yet the scarcity of HRMS reference data, including collision cross-section (CCS) measurements from ion mobility-mass spectrometry (IM-MS) and MS/MS fragmentation spectra, hinders confident structural annotation of chemical exposure agents across laboratories. We therefore developed ToxBase, a multidimensional (*m*/*z*, retention time, CCS, MS/MS) reference database for over 2,000 chemicals sourced from the U.S. Environmental Protection Agency’s ToxCast chemical library. Built via high-throughput liquid chromatography-ion mobility-tandem mass spectrometry (LC-IM-MS/MS), the ToxBase database comprises 3,598 precursor ions spanning 2,075 unique compounds with excellent precision (98.5% of compounds display interday CCS RSDs < 1%) and strong cross-platform agreement. A high-quality MS/MS reference library of the fragmented precursors was assembled using targeted data-dependent acquisition and *DDARawProcessor*, a novel data extraction algorithm. When applied to LC-IM-MS/MS data obtained from human plasma, urine, and fecal samples (n = 20 per matrix), ToxBase rapidly enabled 42 high-confidence (Level 1) identifications. The ToxBase database is freely available and compatible with the open-source MS data processing platform Skyline for vendor-agnostic suspect screening workflows, providing a valuable resource for standardized, large-scale exposome analysis.

## Graphical abstract

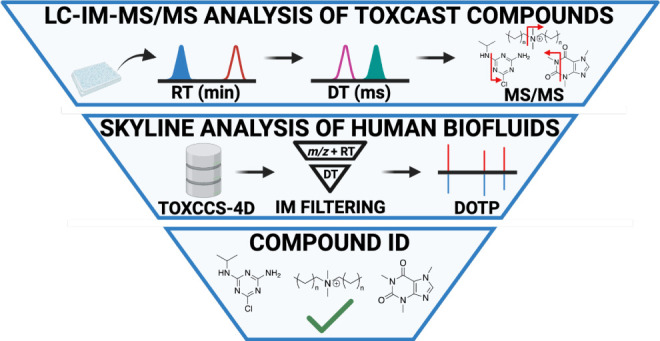

## Introduction

The exposome refers to the complete array of nongenetic factors that an individual experiences during their lifetime.^1–4^ Biological (e.g., viruses, bacteria) and chemical exposures, for example, occur in the context of general environmental factors (e.g., climate, urban environment, socioeconomic status) that may influence or alter biological processes.^5^ Human exposure to complex mixtures of chemicals stems from both voluntary (e.g., disinfectants,^6–9^ pharmaceuticals,^10^ cosmetics^11^) and involuntary sources, such as air pollution^12,13^ and contaminated water.^14,15^ The interactions between these coexposures can lead to synergistic,^16^ antagonistic,^17,18^ and additive^19^ toxic biological effects, which are difficult to predict quantitatively based solely on the toxicity of individual chemicals.^20^ These various mixture effects likely arise from the heterogeneous nature of common environmental chemicals, which span a vast dynamic range in human blood^21^ and represent thousands of distinct classes and structures.^22^ The nongenetic origin of prevalent chronic diseases, including cancer and ischemic heart disease, has become increasingly evident since the completion of the human genome project.^23–26^ Furthermore, recent exposome-wide association studies (ExWAS) have revealed correlations between early life environmental exposures and health outcomes, such as allergies,^27^ childhood obesity,^12^ and all-cause mortality.^28^ The exposome, therefore, represents a crucial component of human health that can provide novel insights into the environmental determinants of chronic diseases and their underlying mechanisms of toxicity.

Significant progress has been made toward accurately measuring the chemical exposome in the modern “omics” era. The routine implementation of high-resolution mass spectrometry, for example, enables researchers to profile broad chemical exposures in complex matrices with high sensitivity and mass accuracy. Interactions between environmental exposures and endogenous processes can now be investigated using a comprehensive multiomics approach^29^ (e.g., adductome, proteome, transcriptome). Longitudinal studies across large, diverse human populations have become more feasible through the development of national^30^ and global^31,32^ research initiatives. Importantly, the Toxicity Forecaster (ToxCast) program has facilitated the identification, prioritization, and toxicological characterization of hazardous chemicals with potential for human and ecosystem exposure.^33^ Since its inception in 2007 by the U.S. Environmental Protection Agency (EPA), the ToxCast chemical library has expanded to include approximately 4,600 unique compounds that span a diverse range of classes^34^ and functions (e.g., pesticides, estrogen and androgen receptor binders, “green” plasticizer alternatives, pharmaceutical agents, and common water contaminants).

Despite these advances, analytical challenges still impose significant limitations on the scope of exposome research. The comprehensive characterization of chemical exposure agents requires broad analytical coverage and sophisticated postacquisition data-processing tools for compound identification.^3^ High-quality reference databases help to fulfill this requirement by providing a powerful tool for confidently annotating mass spectrometry (MS) features. Existing databases such as T3DB^35^ and Exposome-Explorer,^36^ however, do not yet incorporate reference data (e.g., chromatographic information, collision cross-section (CCS) values) that are crucial for unambiguous identification of chemical exposure agents. To address these gaps, we developed ToxBase, a large-scale, multidimensional (*m*/*z*, retention time, collision cross-section, MS/MS) reference database for the ToxCast compounds. While not commonly found in most small molecule reference databases, ion mobility (IM)-derived CCS is a unique physical property of an analyte ion’s gas-phase size and shape. CCS measurement has been shown to be reproducible across different laboratories^6,37–39^ and instrumental platforms.^40–42^ Furthermore, millisecond-scale IM separations interposed between liquid chromatography (LC; seconds) and the mass analyzer (microseconds) add an orthogonal dimension of analyte separation that reduces the MS spectral chimerism arising from multianalyte coelution,^3^ an inherent challenge of complex mixtures. IM-derived CCS measurement, therefore, enables multifactor compound identification when combined with traditional liquid chromatography-tandem mass spectrometry (e.g., LC-IM-MS/MS), which other groups and we have leveraged to increase the confidence of peak annotations for chemical exposure agents in human biofluids.^6,43,44^ In this work, we describe the development of ToxBase, a comprehensive reference database composed of >2,000 unique ToxCast compounds with *m*/*z*, retention time, CCS, and MS/MS spectra, extracted using a home-built Python package, *toxccs*. To demonstrate the advantages of integrating LC-IM-MS/MS workflows into exposome analysis, we profiled human biofluids (e.g., plasma, urine, and feces) using ToxBase coupled with Skyline^45^ and identified ToxCast compounds at different confidence levels. By integrating orthogonal LC, IM, and MS/MS fragmentation reference data for structurally diverse ToxCast compounds, the freely available ToxBase database enables high-throughput suspect screening of chemical exposure agents in complex matrices.

## Materials and Methods

### Materials

Optima LC/MS-grade water, methanol, acetonitrile, and formic acid were purchased from Fisher Scientific (Pittsburgh, PA). Molecular biology-grade dimethyl sulfoxide (DMSO) and poly-_DL_-alanine (PolyAla) were purchased from Sigma-Aldrich (St. Louis, MO). The standard stock solution of PolyAla for ^TWIM^CCS_N2_ calibration was prepared at 25 μg/mL in a 1:1 (v/v) mixture of acetonitrile and water with 0.1% formic acid. Deuterated (*d*_7_-benzyl) benzalkonium chlorides (BACs; *d*_7_-C_10_–C_16_–BACs) were synthesized in-house as previously described.^46^ All 4,684 ToxCast reference standards (0.4 mM in DMSO) were generously provided by Dr. Thomas Metz (Pacific Northwest National Laboratory), diluted into twelve 384-well plates at 10 μM in acetonitrile using automated liquid handling systems at the Quellos High-Throughput Screening Core (University of Washington), and then sealed with prescored silicone mats (Analytical Sales and Services) for long-term storage at −80 °C.

### High-Throughput UPLC-IM-MS Analysis

High-throughput chromatographic separations were performed with a Waters Acquity ultraperformance liquid chromatography (UPLC) system (Waters Corp., Milford, MA), which was equipped with a quaternary solvent pump and refrigerated autosampler (10 °C) coupled to a reversed-phase analytical column (Phenomenex Kinetex, 2.6 μm, polar C18, 100 Å, 30 × 21 mm). Samples (5 μL) were injected and eluted with varying compositions of solvent A (0.1% formic acid in water) and solvent B (0.1% formic acid in methanol) at a flow rate of 0.5 mL/min: 0.00–0.20 min, 100% A; 0.20–0.30 min, 100 → 25% A; 0.30–0.75 min, 25 → 0% A; 0.75–1.05 min, 0% A; 1.05–1.10 min, 0 → 100% A. The eluted analytes were detected on a Waters Synapt XS Q-TOF mass spectrometer in full-scan IM-MS mode over the range of 50–1,200 *m*/*z* using an electrospray ionization (ESI) source and nitrogen as the drift gas. Source parameters for the positive ion (ESI+) and negative ion (ESI−) modes are given in Table S1. Post-IM fragmentation within the transfer region was achieved with a ramp from 25 to 45 V, and the resulting MS^2^ data were stored in a function separate from the MS^1^ acquisition. Note that all fragmentation data used to assemble the ToxBase MS/MS reference library were acquired using a separate data-dependent acquisition (DDA) approach described below to ensure purity of the fragmentation spectra (see High-Throughput UPLC-MS/MS Analysis). Prior to analysis, the mass analyzer was calibrated with sodium formate over the range of 50–1,200 *m*/*z* in “Resolution” mode using IntelliStart. The twelve 384-well plates containing ToxCast reference standards were analyzed in triplicate using both ESI+ and ESI– modes over 12 months to assess interday retention time and CCS variability.

### ^TWIM^CCS_N2_ Calibration

Measured TWIM drift times were calibrated into ^TWIM^CCS_N2_ values using PolyAla reference nitrogen drift tube CCS (^DT^CCS_N2_) values^47^ in both positive and negative ionization modes, as previously described.^6^ Briefly, mobilograms for each singly charged PolyAla calibrant (n = 2–14) were extracted from the raw data with a ±0.01 Da accurate mass window tolerance. Drift times were determined by fitting a Gaussian function to each mobilogram via least-squares regression and then corrected for mass-dependent flight time to yield corrected drift time (*t*_d_′) values. Reference ^DT^CCS_N2_ values were similarly adjusted to account for ion charge state (Z) and reduced mass with nitrogen, producing corrected CCS (CCS′) values. Calibration curves were constructed by fitting corrected drift times and corrected CCS values using the equation:

$$CCS^{\prime }=A{\left({t_{d}}^{\prime }+t_{0}\right)}^{B}$$

where *A*, *t*_0_, and *B* are empirically determined parameters obtained by a nonlinear least-squares regression. To evaluate potential drift time variation that occurred over the course of data acquisition, PolyAla standards were analyzed once at the beginning, middle, and end of each plate, and the resulting measurements (n = 3) were combined to construct a single calibration curve per plate. Calibration performance was assessed by calculating CCS residuals relative to reference values, and calibrators were accepted only when the maximum absolute CCS error was <3%. A representative CCS calibration curve with CCS residuals is shown in Figure S1.

### High-Throughput UPLC-MS/MS Analysis

A data-dependent acquisition (DDA) approach was used to assemble the ToxBase MS/MS reference library. Following triplicate UPLC-IM-MS analysis, the twelve 384-well plates were reinjected and analyzed on the same Synapt G2-XS Q-TOF instrument using the chromatographic gradient and instrument parameters described above, but with IMS off (Table S1) in both ESI+ and ESI– ionization modes. The mass analyzer was calibrated with sodium formate over the range of 50–1,200 *m*/*z* in “Resolution” mode using IntelliStart prior to analysis. Additionally, a leucine-enkephalin lock mass ([M+H]^+^ = 556.2771; [M–H]^−^ = 554.25) was directly infused into the ion source at the beginning and end of each acquisition for real-time corrections of mass shifts. Detailed instrument conditions and parameters are provided in Table S2.

### UPLC-IM-MS Data Processing

A previously described UPLC-IM-MS data processing workflow was adapted to develop *toxccs*, a Python package designed to efficiently process and visualize the raw data.^6^ First, target lists containing the theoretical *m*/*z* values of common ESI adducts ([M+H]^+^, [M+Na]^+^, [M+K]^+^, [M+H–H_2_O]^+^, [M–H]^−^, [M+Cl]^−^, [M+FA–H]^−^, and [M–H–H_2_O]^−^) were used to automatically extract *m*/*z*-selected LC ion chromatograms (EICs) from raw UPLC-IM-MS data files with a mass tolerance of ±0.025 Da. Local maxima (retention times) and peak boundaries in the EIC were detected using the “find_peaks” function in SciPy (version 1.8.1).^48^ Extracted ion mobility traces (EIMs), also referred to as mobilograms, were subsequently constructed by expanding the *m*/*z* and retention time window feature in the mobility dimension and fitting the data with a Gaussian function. Features in the EIM were subsequently detected to obtain drift times, which were automatically converted to calibrated ^TWIM^CCS_N2_ values by applying the CCS calibration curve described above (see ^**TWIM**^ **CCS**_**N2**_ Calibration). Extracted peaks exhibiting low intensity (<1e3) and/or mass error >10 ppm were removed, and the entire dataset (>25,000 features) was manually inspected for peak shape and quality of the Gaussian fit. All data processing was performed with *toxccs*, a comprehensive Python package developed in-house that directly interfaces with Waters UPLC-IM-MS data files in their native (.raw) format. The *toxccs* package and all code for generating the ToxBase reference database are freely available on GitHub (https://github.com/libinxulab/xulab_software.git).

### UPLC-MS/MS Data Processing

A collection of over 5,000 MS/MS fragmentation spectra was extracted from raw UPLC-MS/MS data files with *DDARawProcessor*, a *toxccs* module designed specifically for DDA files. To increase the throughput of identifying and exporting the optimal MS^2^ scan for a given feature, a novel algorithm was developed, wherein candidate scans in various function numbers are automatically compared and evaluated. Accepted features from the UPLC-IM-MS analysis (see UPLC-IM-MS Data Processing) were assembled into target lists to construct the MS^1^ survey EIC. MS^2^ scans were then extracted by expanding the *m/*z and retention time window feature in all MS^2^-level function numbers and then ranked by precursor *m*/*z* intensity. The identified MS/MS fragmentation spectra were exported to Excel spreadsheets for the database assembly.

### Assembly of ToxBase Reference Database

We generated a multidimensional reference database in the mass spectral library (MSP) file format. As described above (see UPLC-IM-MS Data Processing), retention times and calibrated ^TWIM^CCS_N2_ values were automatically extracted from raw UPLC-IM-MS data files and manually reviewed. The reported ^TWIM^CCS_N2_ and retention time values for each database feature (i.e., compound/adduct pair) represent the average measurement (n = 3) of technical replicates. Features that displayed a ^TWIM^CCS_N2_ relative standard deviation (RSD) > 3% were removed from the database. To assemble the ToxBase database, a custom Python script was used to merge the following data into a single text file consisting of compound name, precursor theoretical *m*/*z*, adduct type, chemical formula, retention time, ^TWIM^CCS_N2_, and MS/MS fragmentation spectrum as a peak list of *m*/*z* and intensity values. Finally, the text file was converted to the MSP format for direct compatibility with Skyline. The keys for each field in the ToxBase reference database are defined in Table S3.

### Human Sample Collection

Blood, urine, and fecal samples were collected from healthy volunteers between the ages of 18 and 49 years with a BMI of 18 to 27 kg/m^2^. The study was approved by the University of Washington Institutional Review Board (under STUDY00013949), and all participants completed an online screening questionnaire with the following exclusion criteria: taking medication or supplements within the last 2 weeks that could affect cholesterol or lipid levels; diagnosis of any significant medical conditions, including hepatic, renal, cardiac, gastrointestinal, or autoimmune disease, diabetes, or other metabolic diseases; or currently pregnant or breastfeeding. All participants gave informed consent. Consented participants received a commercially available fecal collection kit (EasySampler Stool Collection Kit, ALPCO, Salem, NH) and were instructed to collect fecal samples within 24 h of their scheduled visit. Samples were aliquoted into four screw-cap tubes (∼2 mL each), placed in sealable bags, and stored in home freezers until the visit. Participants also fasted overnight (no food consumption after midnight, water permitted) and arrived between 8:30 and 10:30 am the following morning. At the visit, participants collected urine into sealed specimen containers (Starplex Scientific, Ontario, Canada), from which ∼14 mL was aliquoted into seven labeled microcentrifuge tubes (Fisher Scientific, Hampton, NH). Approximately 14 mL of blood was drawn into a Vacutainer EDTA tube (Becton Dickinson, Franklin Lakes, NJ) and centrifuged at 1,000 *g* for 10 min at 4 °C. Plasma, white blood cell, and red blood cell fractions were separated and transferred into labeled microcentrifuge tubes. All collected samples were stored at −80 °C until analysis.

### Preparation of Human Plasma Samples

Plasma samples (n = 20) were thawed at room temperature and vortexed to ensure homogenization. For individual samples, 30 μL ice-cold acetonitrile containing a mixture of deuterated benzalkonium chlorides (BACs; 0.3 pmol each of *d*_7_-C_10_–C_16_–BACs) was added, followed immediately by 10 μL plasma. The tubes were then gently vortex-mixed, placed on ice for 15 min to allow protein precipitation, and centrifuged at 16,000 *g* for 15 min at 4 °C. Following centrifugation, 20 μL supernatant was transferred into glass autosampler vials containing 40 μL of a 2:1 (v/v) mixture of water and methanol. The vials were capped with a preslit cap and gently vortexed prior to analysis. Blank water controls (extraction of 10 μL water in place of plasma) were prepared in quintuplet alongside the samples using the same solvents, pipettes, and tubes.

### Preparation of Human Urine Samples

Urine samples (n = 20) were thawed at room temperature, vortexed, and transferred (100 μL) to microcentrifuge tubes. For extraction, 100 μL ice-cold acetonitrile containing the internal standards (2.5 pmol each of *d*_7_-C_10_–C_16_–BACs) was added. Samples were then vortex-mixed and centrifuged at 16,000 *g* for 10 min at 4 °C. Finally, the supernatant (100 μL) was transferred to glass autosampler vials containing 100 μL water. Procedural blanks (n = 5) were prepared by substituting 100 μL water for urine.

### Preparation of Human Fecal Samples

Fecal samples (n = 20) were prepared as previously described.^6^ Briefly, samples (50 mg wet weight) were weighed on ice and then dispersed in 200 μL of a 3:1 water/ethanol solvent mixture by vortex mixing and sonication. The resulting mixtures were spiked with 20 μL acetonitrile containing the internal standards (10 pmol each of *d*_7_-C_10_–C_16_–BACs), precipitated with acetonitrile, and then centrifuged at 16,000 *g* (4 °C). Supernatants were recovered prior to re-extraction with a solution of 9:1 methanol/chloroform. After combining the supernatants and evaporating them to dryness overnight, the residues were reconstituted in 4:1 acetonitrile/water, filtered through 0.22 μm spin filter tubes, and transferred to glass autosampler vials for UPLC-IM-MS^E^ analysis. Procedural blanks (n = 5) were prepared by substituting 50 μL of water for feces and processed identically.

### UPLC-IM-MS^E^ Analysis of Human Biofluid Samples

Human biofluid extracts (5 μL) were injected and separated with the UPLC-Q-TOF system, chromatographic gradient, and instrument source parameters described above (see High-Throughput UPLC-IM-MS/MS Analysis and Table S1) in the ESI+ mode. To maximize the collection of both precursor and fragment ion data, the eluted analytes were detected in full-scan IM-MS^E^ (HDMS^E^) mode over the range of 50–1,200 *m*/*z* using the instrument conditions described in Table S4. Prior to analysis, the mass analyzer was calibrated with sodium formate over the range of 50–1,200 *m*/*z* in “Resolution” mode using IntelliStart. A leucine-enkephalin lock mass ([M+H]^+^ = 556.2771) was also directly infused into the ion source at the beginning and end of each acquisition for real-time corrections of mass shifts. The raw UPLC-IM-MS^E^ data files were directly imported into Skyline (version 24.1.0), lock mass-corrected, and then automatically processed after importing the reference retention times, ^TWIM^CCS_N2_ values, and MS/MS fragmentation spectra in the ToxBase MSP library. An initial list of putative annotations was obtained by removing features present in procedural blanks. This background-filtered list was then manually reviewed in Skyline to assess peak quality and monitor method stability by evaluating shifts in retention time, mass accuracy, and peak intensity of the spiked deuterated benzalkonium chlorides (*d*_7_-C_10_–C_16_–BACs) added to each biofluid sample. These data are summarized in Table S5 and the Supporting Information (see Instrument Stability). Matrix effects and recoveries for a mixture of 8 native ToxCast standards were also evaluated in each matrix by comparing the peak areas of samples spiked pre- and postextraction to those of equivalent standards prepared in neat solvent (see Analytical Performance in the Supporting Information). Matrix effects and recoveries were generally within ±20% with the exception of DEET in feces, which displayed low recovery (<1%) but relatively minor (∼15%) matrix effects, indicating that the compound was likely lost during sample preparation rather than suppressed during ionization.

For each precursor, MS/MS agreement was evaluated by comparing the observed fragment ion pattern to the corresponding ToxBase reference spectrum. This comparison was standardized across samples by selecting the top 3 mobility-aligned product ions per precursor (based on reference library peak intensity ranking). Spectral similarity was assessed using Skyline’s dot product score (dotp), which reports the similarity between observed transition peaks and the reference library spectrum (range 0–1). Similarity between observed and theoretical isotopic distribution patterns was also assessed for each precursor using Skyline’s isotopic distribution score (idotp). Threshold values of 0.7 for idotp and 0.5 for dotp were selected to remove low-quality matches. IM filtering was implemented in Skyline using a fixed mobility window of 0.3 ms, centered on the reference drift time value. Finally, features were excluded if they displayed retention time differences greater than 0.1 min relative to the reference retention time recorded in ToxBase. For a detailed description of annotation thresholds and confidence levels used for compound identification, see Table S6.

### Code and Data Availability

The code for raw data processing and visualization is freely available on GitHub as *toxccs*, a Python package that directly interfaces with native DIA (HDMS^E^) and DDA file formats (https://github.com/libinxulab/xulab_software.git). Experimental raw data files used to generate the ToxBase reference database are available on MassIVE (MSV000100038; doi: 10.25345/C5GB1XW6D). The ToxBase reference database is freely accessible as a Microsoft Excel spreadsheet in the Supporting Information. Skyline files and the associated raw experimental data, as well as the ToxBase MSP library, are available on Panorama Public (https://panoramaweb.org/yMRzll.url).

## Results and Discussion

### High-Throughput UPLC-IM-MS Analysis

This study encompassed compounds from the U.S. EPA’s ToxCast library, a structurally and functionally diverse collection of pesticides, flame retardants, plasticizers, color dyes, cosmetic ingredients, disinfectant products, and other common environmental pollutants with potential for human and ecosystem exposure.^49^ A high-throughput analytical workflow, shown in Figure 1, was developed to rapidly measure retention times and ^TWIM^CCS_N2_ values and to obtain MS/MS fragmentation spectra for the ToxCast reference standards. The total analysis time per well was approximately 2 min, enabling high-throughput analysis of individual reference standards across two 384-well plates within a 24-h period.

**Figure 1:**
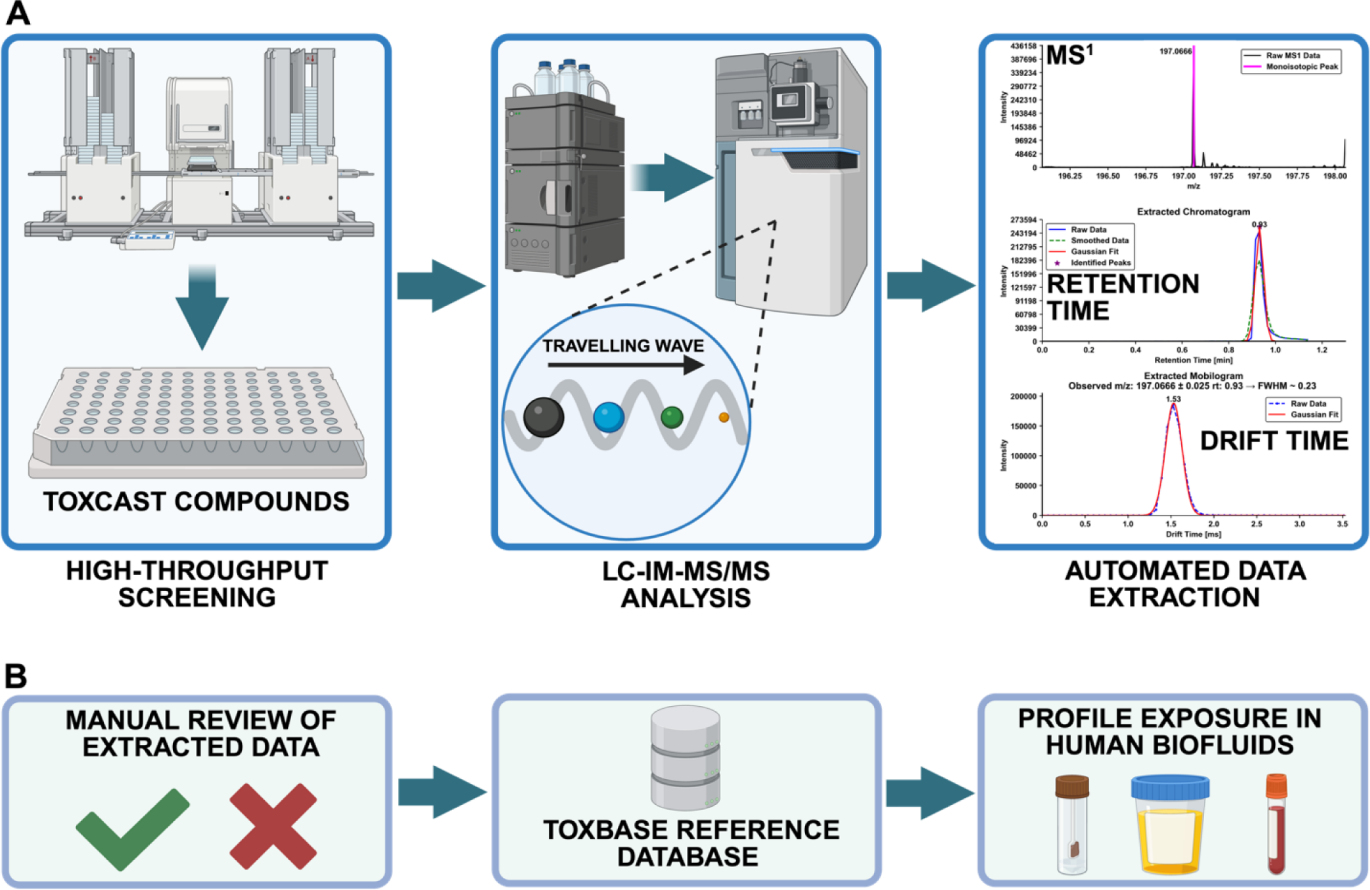
Overview of the experimental workflow for assembling ToxBase. (A) ToxCast reference standards were diluted in acetonitrile (10 μM) using automated liquid handling platforms at the Quellos High-Throughput Screening Core (University of Washington) and subsequently analyzed via LC-IM-MS. The raw data files were automatically processed to extract precursor *m*/*z* values, retention times, and drift times (from mobilograms), which were subsequently calibrated into ^TWIM^CCS_N2_ measurements using the *toxccs* Python package. (B) Extracted data were manually reviewed for peak shape and quality of the Gaussian fit prior to assembling the ToxBase reference database, which was merged with the MS/MS reference library acquired using a data-dependent acquisition (DDA) approach (see High-Throughput UPLC-MS/MS Analysis). The multidimensional ToxBase reference database, containing *m*/*z* values, retention times, CCS measurements, and MS/MS spectra, was utilized to profile plasma, urine, and fecal samples from healthy donors.

To increase the throughput of our analytical workflow, we developed a Python package, *toxccs* (https://github.com/libinxulab/xulab_software.git), that interfaces directly with UPLC-IM-MS data files in the native (.raw) format. This package was utilized to automatically extract retention times and calibrated ^TWIM^CCS_N2_ values for the ToxCast reference standards included in our study (see UPLC-IM-MS Analysis in Materials and Methods and Figure 1). Approximately 28,000 individual data files, totaling over 6 TB, were generated from triplicate analyses of each reference standard (ESI+ and ESI– modes) and subsequently processed with *toxccs*. The resulting dataset was then manually reviewed for quality control of automated Gaussian fits.

Collectively, 3,598 ions representing 2,075 unique chemicals (44.3% of the 4,684 analyzed ToxCast standards) were detected. In the positive ion mode, 2,346 cations (1,111 [M+H]^+^, 47 [M]^+^, 739 [M+Na]^+^, 148 [M+K]^+^, 301 [M+H–H_2_O]^+^) were detected (Figure S2A), while a total of 1,252 anions (884 [M–H]^−^, 1 [M]^−^, 61 [M+Cl]^−^, 240 [M+FA–H]^−^, and 66 [M–H–H_2_O]^−^) were detected in the negative ion mode (Figure S2B). Of the 2,075 unique chemicals represented in the ToxBase reference database, 842 (40.6%) belong to the benzenoid superclass using ClassyFire,^34^ reflecting the high prevalence of aromatic environmental contaminants. Organoheterocyclic (20.1%), lipid and lipid-like molecules (11.6%), and organic acids and derivatives (10.7%) also constitute large portions of the structurally diverse database, which encompasses 16 distinct superclasses and 159 classes, as shown in Figure S2C (6 compounds could not be classified at these levels). ToxCast compounds for which ^TWIM^CCS_N2_ values were not successfully determined in either ESI+ or ESI– modes include prenol lipids, hydrocarbons, and other classes more amenable to alternative ionization sources and/or techniques (e.g., inductively coupled plasma-MS and gas chromatography electron ionization). These results are consistent with a recent study by Teri et al., who determined the drift tube ^DT^CCS_N2_ values for 2,140 (∼45.7%) of the ToxCast library using ESI+, ESI–, and atmospheric pressure chemical ionization (APCI) sources.^50^ Notably, these authors found chemical stability to be strongly associated with detectability via IM-MS. However, this previous database lacks the retention time and MS/MS dimensions currently available in ToxBase.

### Assembly of the ToxBase MS/MS Reference Library

To further increase confidence levels in exposome analysis, a high-quality MS/MS reference library was assembled from the ToxCast reference standards (see High-Throughput UPLC-MS/MS Analysis and UPLC-MS/MS Data Processing in Materials and Methods). Spectral library matching is a crucial step in MS-based compound identification workflows and can significantly reduce the occurrence of false annotations, especially in the absence of CCS.^6,51–57^ The methods for acquiring MS/MS spectra vary in both the throughput and complexity. Targeted analysis (e.g., multiple reaction monitoring) involves the fragmentation of user-defined precursor ions, yielding highly specific and unambiguous MS/MS spectra. This approach, however, is time-consuming and therefore not ideal for large collections of reference standards, as each compound typically requires prior optimization in a separate injection or dedicated experiment. In contrast, data-independent acquisition (DIA) methods (e.g., MS^E^) sample all precursor ions across wide *m*/*z* values and support high-throughput analysis of complex mixtures. Because the resulting MS/MS spectra must be computationally deconvoluted following acquisition,^58^ DIA methods are not always practical for building large-scale reference libraries. Furthermore, the simultaneous fragmentation of coeluting precursor ions in DIA methods can introduce interfering ions from unrelated species at the same drift time, reducing spectral purity and increasing the likelihood of ambiguous or incorrect compound identifications.

A data-dependent acquisition (DDA) approach was therefore used to assemble the ToxBase MS/MS reference library. Unlike DIA and targeted methods, DDA employs a narrow precursor isolation window to rapidly obtain clean, unambiguous fragmentation spectra for sample components without prior experimental optimization. Although DDA collects MS/MS data for only a subset of detected ions, the use of concentrated reference standards in building the ToxBase library enabled broad coverage of the targeted compounds. In total, MS/MS fragmentation spectra for 1,629 compounds (∼79% of the acquired ToxBase database) were recorded. Although several commercial and open-source platforms support MS/MS spectral library searching and annotation, to the best of our knowledge, similar tools are not available for automated *extraction* of MS/MS spectra from Waters .raw DDA files. We therefore developed *DDARawProcessor*, a *toxccs* module designed in-house to address the paucity of open-source tools for small molecule MS/MS library development. The module was implemented to extract, rank, and export the most ideal MS/MS scan for target precursor ions by iterating through all nonsurvey scan functions containing fragmentation data. After carefully evaluating the resulting data (spectra lacking peaks with intensity > 1e3 were excluded), the final ToxBase MSP library was assembled by linking features identified by UPLC-IM-MS to their extracted MS/MS spectra.

### IM-MS Conformational Space of ToxCast Compounds

The correlation between ^TWIM^CCS_N2_ and *m*/*z* was best described by the power regression model (*R* ^2^ value of 0.87) as depicted in the ^TWIM^CCS_N2_ versus *m*/*z* (i.e., IM-MS conformational space plot) for the 3,598 detected ions (Figure 2A).

**Figure 2:**
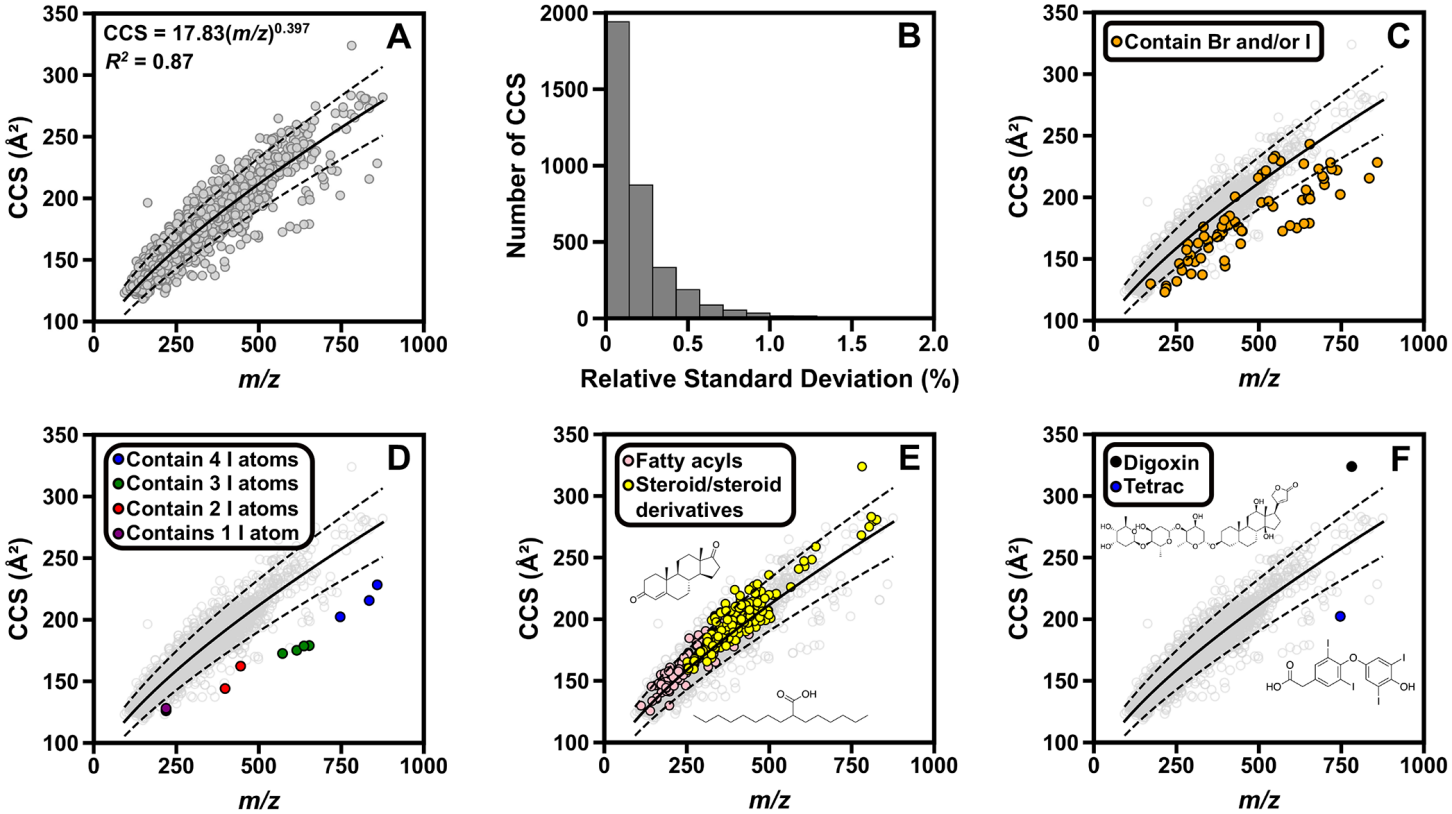
(A) IM-MS conformational space plot displaying the 3,598 ^TWIM^CCS_N2_ values of the 2,075 detected ToxCast compounds in ToxBase. (B) Histogram of the relative standard deviations (RSDs) of the triplicate ^TWIM^CCS_N2_ measurements. IM-MS conformational space plot highlighting (C) compounds with bromine and/or iodine atoms (orange), (D) compounds with 1–4 iodine atoms (purple, red, green, and blue, respectively), (E) compounds classified^34^ as fatty acyls (pink) and steroid/steroid derivatives (yellow), and (F) digoxin (black) and tetrac (blue). In all IM-MS conformational space plots, the central power trendline and ±10% bands are displayed as solid and dashed lines, respectively.

Triplicate ^TWIM^CCS_N2_ measurements were highly reproducible, with the majority (98.5%) of values displaying interday relative standard deviations (RSDs) less than 1% (Figure 2B). The ^TWIM^CCS_N2_ values span a 2.73-fold range from 118.39 Å^2^ (trifluoromethanesulfonic acid; [M–H]^−^) to 323.94 Å^2^ (digoxin; [M+H]^+^). The *m*/*z* values span a 9.41-fold range from 93.0346 Da (phenolate; [M–H]^−^) to 875.4877 Da (roxithromycin; [M+K]^+^). Figure 2A illustrates the distribution of measured ^TWIM^CCS_N2_ values that fall within the region ±10% (dashed lines) from the central power trendline (solid line). Although the majority of compounds (93.2%) fall within the ±10% bounds, there are outliers (246 ions) that clearly illustrate the structural diversity and unique gas-phase trends in ToxBase. Compounds with one or more bromine or iodine atoms, for example, consistently fell below the central power trendline (Figure 2C). As we have previously described,^39^ these molecules exhibit higher gas-phase densities and smaller ^TWIM^CCS_N2_ values compared to compounds of similar mass, as heavy halogen atoms significantly increase molecular mass without proportionally increasing molecular size. Several organoiodine compounds formed distinct clusters in IM-MS space according to the number of iodine atoms present (Figure 2D). These compounds—which include food additives (FD&C Red 3, erythrosin B), thyroid hormone analogues (tetrac), radiological contrast agents (diatrizoate, iopanoic acid), and antibacterial agents (iodoquinol, diiodomethyl 4-methylphenyl sulfone)—also generally clustered according to their respective bioactivities, illustrating the potential relationship between chemical function and gas-phase structural characteristics.

Compounds within the ±10% bounds also displayed structure-dependent relationships between ^TWIM^CCS_N2_ and mass. Different classes of lipid and lipid-like molecules, for example, were found to occupy distinct regions of IM-MS space, as evident in Figure 2E. While both fatty acyls and steroid/steroid derivatives generally exhibited decreased gas-phase density compared to similar-mass ions, we observed that molecules based on the cyclopenta­[a]­phenanthrene carbon skeleton tended to deviate less from the central power trendline (69.7% steroid/steroid derivatives above the trendline) than fatty acyls with hydrophobic chains (76.9% of fatty acyls above the trendline). Diversity within each class was also observed. For example, malaoxon (315.0662 Da; 161.37 Å^2^; [M+H]^+^) and octyl beta-d-glucopyranoside (315.1778 Da; 179.52 Å^2^; [M+Na]^+^) have a mass percent difference of only 0.04% but a difference of 10.6% in ^TWIM^CCS_N2_ measurements. The structural diversity in ToxBase is further illustrated by comparing compounds above 600 Da, where particularly large differences in measured ^TWIM^CCS_N2_ were observed between digoxin (781.4369 Da; 323.94 Å^2^; [M+H]^+^) and organohalogens such as tetrac (746.6528 Da; 202.27 Å^2^; [M–H]^−^) and erythrosin B (834.6478 Da; 215.56 Å^2^; [M–H]^−^) (Figure 2F). The large differences in ^TWIM^CCS_N2_ are likely driven by a combination of factors, including ion-neutral interactions between digoxin’s numerous hydroxyl groups and the drift gas.^59^

Interestingly, a large number of compounds (101) exhibited bimodal mobilograms from which two ^TWIM^CCS_N2_ measurements were determined for a single *m*/*z* value and retention time window (see **ToxBase** in the Supporting Information for a complete list). This phenomenon, which we and others have reported,^39,60,61^ may stem from the presence of isomers or protomers forming unique gas-phase conformations in the drift tube. Compounds with more than one major mobilogram peak were primarily substituted benzenoids (30%) and steroids/steroid derivatives (21%), suggesting that multiple gas-phase conformations could be a predictable feature among certain structurally related molecules. While some cases of bimodal mobilograms, such as members of the fluoroquinolone and β-lactam antibiotic classes, have previously been reported,^39^ several new cases were identified that demonstrate the richness and complexity of IM-MS datasets. For example, etoposide (Figure 3A)—a beta-d-glucoside DNA synthesis (topoisomerase II) inhibitor—exhibited a single peak in the LC dimension (Figure 3B) but two major mobilogram peaks in the ESI– mode (Figure 3C).

**Figure 3:**
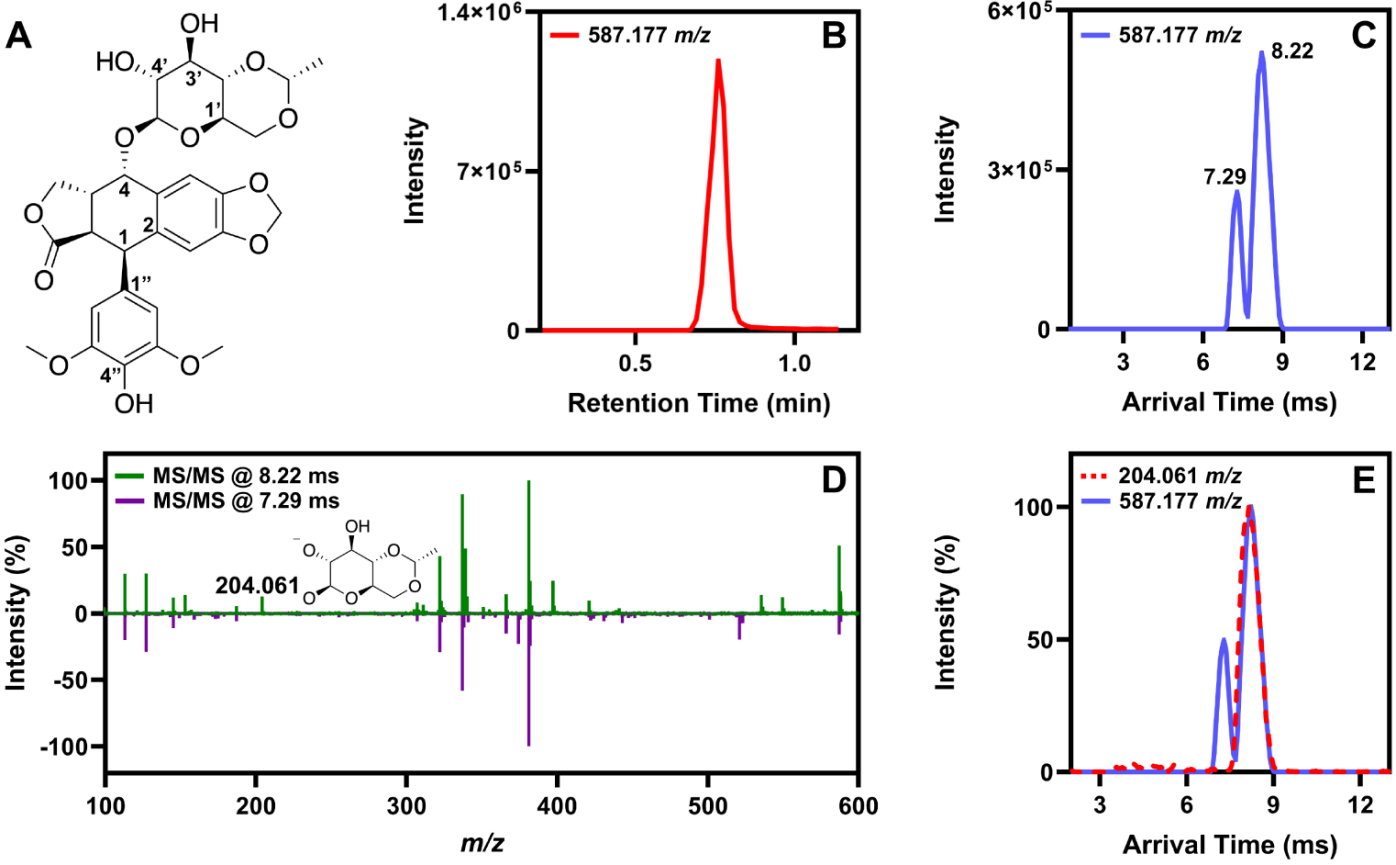
(A) Chemical structure of etoposide. (B) LC-MS extracted ion chromatogram and (C) bimodal IM-MS extracted ion mobilogram displaying the [M–H]^−^ adduct of etoposide (±0.025 Da from theoretical *m*/*z* value). (D) Mirror plot displaying post-IM fragmentation spectra extracted at the apex of the earlier-arriving mobilogram peak (purple) and the later-arriving mobilogram peak (green) of etoposide. (E) IM-MS extracted ion mobilogram (±0.025 Da from theoretical *m*/*z* value) displaying the [M–H]^−^ adduct of etoposide (solid blue) and 204.06 *m*/*z* fragment (dashed red), corresponding to the deprotonated ethylidene glucoside moiety observed exclusively in the later-arriving mobilogram peak.

To further investigate the structural basis of the gas-phase separation, post-IM fragmentation spectra were obtained by selectively applying energy to the transfer region of the mass spectrometer (see High-Throughput UPLC-IM-MS Analysis in Materials and Methods). Distinct fragmentation patterns were observed at the apex of each EIM, as shown in Figure 3D. A unique fragment ion corresponding to the deprotonated ethylidene glucoside moiety on C4 was observed in the later-arriving EIM, indicating deprotonation at either the C3′ or C4′ hydroxy group. In contrast, the fragmentation spectrum acquired for the earlier-arriving EIM is consistent with deprotonation at the C4″ hydroxy group. Figure 3E further demonstrates the selectivity of the 204.06 *m*/*z* mobility-aligned fragment, which is exclusively present in the later-arriving EIM peak.

### Comparison to Literature CCS Values

To evaluate the interlaboratory and interplatform reproducibility of the ^TWIM^CCS_N2_ data, we compared our results to ^DT^CCS_N2_ values recently published by Teri et al.^50^ CCS differences (ΔCCS %) for [M+H]^+^, [M+Na]^+^, and [M–H]^−^ adducts are displayed in Figure 4.

**Figure 4:**
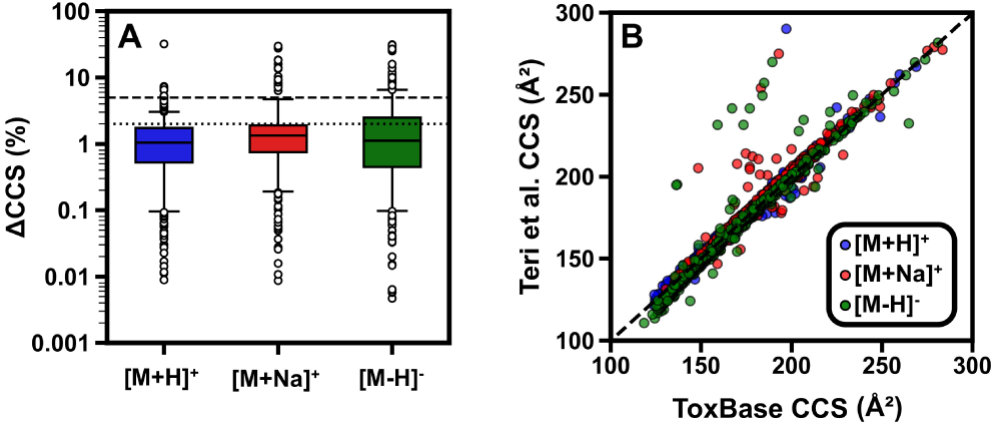
(A) Comparison of ^TWIM^CCS_N2_ values for [M+H]^+^ (blue), [M+Na]^+^ (red), and [M–H]^−^ (green) adducts in ToxBase with ^DT^CCS_N2_ reported by Teri et al.^50^ Boxes indicate the interquartile range and median (solid horizontal line), with whiskers spanning the 5th to 95th percentiles and outliers shown as individual points. The dotted and dashed horizontal lines mark the discussed 2% and 5% thresholds, respectively. (B) Pairwise scatter plot of ToxBase ^TWIM^CCS_N2_ values versus ^DT^CCS_N2_ values reported by Teri et al. Each point represents a compound-adduct pair, colored by adduct type. The dashed black line (y = x) represents perfect agreement between the datasets.

A total of 1,592 CCS values (595 [M+H]^+^, 472 [M+Na]^+^, and 525 [M–H]^−^ adducts) representing 1,128 unique ToxCast compounds were found in both databases and compared. CCS differences within 2% are generally considered acceptable to account for instrument variability.^62^ The median CCS differences for the [M+H]^+^, [M+Na]^+^, and [M–H]^−^ adducts were 0.87%, 1.26%, and 0.83%, respectively, with 80% of the CCS values exhibiting differences within 2% across instrumental platforms (Figure 4A). Among the ToxBase compounds with a single ^TWIM^CCS_N2_ value, only 47 (4.2%) showed CCS differences greater than 5%—a threshold previously established to reflect experimental differences (e.g., CCS calibration, electric field method, and pressure or temperature fluctuations within the drift tube).^50,62^ These results are consistent with previous interlaboratory assessments of CCS suspect screening libraries.^50,62^

CCS reproducibility may be influenced by a number of factors. As discussed above (see High-Throughput UPLC-IM-MS Analysis in Materials and Methods), compounds with multiple protonation sites can form conformationally distinct protomers in the gas phase upon ionization, resulting in the determination of multiple CCS values. These protomers may not be resolved in instruments with lower mobility resolution, leading to discrepancies across platforms and in reported data.^62^ For example, candesartan cilexetil—an angiotensin-receptor blocker prodrug—exhibited a bimodal mobilogram in the ESI+ mode, resulting in two calculated ^TWIM^CCS_N2_ values (224.97 Å^2^ and 241.15 Å^2^). While the ^DT^CCS_N2_ value reported by Teri et al. (242.2 Å^2^) closely matched our larger ^TWIM^CCS_N2_ value (ΔCCS = 0.43%), the presence of a second, earlier arriving protomer in the TWIM data clearly explains the high observed CCS difference for candesartan cilexetil (ΔCCS = 7.1% between 224.97 Å^2^ and 242.2 Å^2^). Interestingly, 8 of the 20 compounds with the highest ΔCCS exhibited bimodal mobilograms, wherein one of the two measured ^TWIM^CCS_N2_ values aligned closely with the corresponding ^DT^CCS_N2_ value (see **Multiple DT Peaks** in the Supporting Information for a complete list of compared compounds). For these cases, comparison of the second ^TWIM^CCS_N2_ value in our database with Teri et al. yielded large differences. As illustrated in Figure 4B, many of these compounds appear significantly above the general y = x trendline, highlighting the potential for bimodal peak behavior to influence interplatform CCS comparisons. Overall, the strong agreement between TWIM and DT data demonstrates the suitability of CCS for identifying structurally diverse chemical exposure agents, but caution should be taken for ions with multimodal conformations.

In addition to conformational heterogeneity, interplatform CCS reproducibility may also be influenced by the effects of external calibration. While previous studies comparing ^TWIM^CCS, ^DT^CCS, and trapped ion mobility spectrometry (^TIMS^CCS) measurements for lipids^63^ and steroids^64,65^ have reported generally good agreement with differences within 1–2%, the calibration step is known to be important for reproducible CCS determination between MS platforms. For example, in their comparison of plasma lipid CCS measurements, George et al. observed a range of mean RSDs (∼0.5–3%) between ^DT^CCS, ^TWIM^CCS, and ^TIM^CCS using different sets of external calibrants,^63^ although it should be noted that the highest RSDs were associated with inadequate coverage of lipid *m*/*z* and CCS ranges. Overall, reproducible CCS values (mean RSDs ∼ 1%) were determined on TWIM and TIMS instruments using identical sets of appropriate calibrants. Although large-scale comparisons between commercial IM-HRMS technologies for structurally diverse small molecules remain limited, the present study and prior cross-platform investigations collectively support that CCS measurements are broadly comparable across IM platforms, supporting the use of CCS as an orthogonal identification parameter in suspect screening workflows.

ToxBase includes CCS values for 765 unique compounds and 1,376 CCS values not reported by Teri et al., spanning a wide range of chemical superclasses and functions. Of these, 289 (37.8%) are benzenoids representing environmental pollutants and consumer ingredients—for example, triclosan (a personal-care antimicrobial used extensively prior to being banned by the U.S. FDA in 2016)^66^ and the widely applied synthetic pesticides chlorpyrifos^67,68^ and potassium 1-naphthaleneacetate.^69^ ToxBase also contains novel reference data for compounds that have been detected with high frequency in human populations, including the UV light filter 4-hydroxybenzophenone^70^ and 2-naphthylamine, a carcinogenic aromatic amine found in tobacco smoke and various consumer products (e.g., hair dyes, food colorants, kitchenware).^71–74^ These data provide expanded coverage of chemical exposure agents in compound identification workflows.

### Exposomic Analysis of Human Plasma, Urine, and Fecal Samples

To demonstrate the utility of ToxBase in exposome analysis, we profiled plasma, urine, and fecal samples from healthy human donors (n = 20 per matrix). Following sample extraction and data acquisition (see **UPLC-IM-MS** **E** **Analysis of Human Plasma Samples**), raw UPLC-IM-MS^E^ files were directly imported into Skyline along with the reference retention times, ^TWIM^CCS_N2_ values, and MS/MS fragmentation spectra in the ToxBase MSP library. Features detected in procedural blanks were removed prior to assigning a confidence level to each putative structure in the filtered dataset (Table S6). The confidence levels—first defined by Schymanski et al.^75^ and recently adapted by Boatman et al.^76^ for PFAS identification in IM-MS nontargeted analyses—were as follows: **Level 1 (Confirmed Structure),** a single structure verified by analysis of an authentic reference standard under identical conditions, with exact *m*/*z*, isotopic pattern, CCS, and retention time or retention index agreement, plus supporting mobility-aligned fragments; **Level 2 (Probable Structure),** a single structure with sufficient supporting evidence to exclude alternatives, meeting *m*/*z*, isotopic pattern, CCS, and retention time or retention index matches to a library or predicted values, plus supporting mobility-aligned fragments; **Level 3 (Tentative Candidate Structures),** one or more candidates consistent with all experimental evidence (*m*/*z*, isotopic pattern, CCS, and retention time or retention index matches to library or predicted values for all candidates), with supporting mobility-aligned fragments. Because these studies served as proof-of-concept for compound identification using the multidimensional ToxBase, no concentration was determined. The reported compounds and their detection frequencies should, therefore, be interpreted as qualitative identifications intended solely for method demonstration. It should also be noted that matrix-dependent ionization effects and extraction efficiencies can influence the detectable chemical space of a given analysis, and comprehensive evaluation of these factors represents an important consideration for future applications of ToxBase.

A total of 42 unique ToxCast compounds were detected across the 3 matrices and identified with Level 1 confidence (Table S7). Identified compounds (n = 22) that displayed a detection frequency >75% in at least one matrix are listed in Table 1.

**Table 1:** High-Frequency (>75%) Level 1 Identifications across Human Plasma, Urine, and Feces (N = 20)^a^

Candidate Structure	Formula	Classification	DF (%) (Matrix)
1,2-Dimethyl-5-nitroimidazole	C_5_H_7_N_3_O_2_	Pesticide	85 (Plasma)
2′-Aminoacetophenone	C_8_H_9_NO	Food Additive	90 (Feces)
4-Anilinophenol	C_12_H_11_NO	Pesticide	100 (Urine) 85 (Feces)
4-Dimethylaminobenzaldehyde	C_9_H_11_NO	Chemical Industrial	95 (Feces)
4-Nitrosodiphenylamine	C_12_H_10_N_2_O	Chemical Industrial	85 (Urine)
5-Ethyl-2,3-pyridinedicarboxylic acid	C_9_H_9_NO_4_	Chemical Industrial	100 (Urine)
8-Hydroxyquinoline	C_9_H_7_NO	Cosmetic Ingredient	95 (Plasma) 95 (Urine) 100 (Feces)
Ancymidol	C_15_H_16_N_2_O_2_	Pesticide	85 (Feces)
Benzalkonium C14	C_23_H_42_N^+^	Cosmetic Ingredient	100 (Feces)
Benzo(f)quinoline	C_13_H_9_N	Polycyclic Aromatic Hydrocarbons	85 (Urine)
Benzylhexadecyldimethylammonium	C_25_H_46_N^+^	Cosmetic Ingredient	95 (Feces)
Decamethylcyclopentasiloxane	C_10_H_30_O_5_Si_5_	Cosmetic Ingredient	100 (Urine)
Deethylatrazine	C_6_H_10_ClN_5_	Pesticide	75 (Plasma)
Deisopropylatrazine	C_5_H_8_ClN_5_	Pesticide	75 (Urine)
Didecyldimethylammonium	C_22_H_48_N^+^	Cosmetic Ingredient	85 (Feces)
Ethylhexadecyldimethylammonium	C_20_H_44_N^+^	Cosmetic Ingredient	95 (Urine) 80 (Feces)
Lithocholic acid	C_24_H_40_O_3_	Bile Acids and Derivatives	90 (Feces)
*N*-Phenyl-1,4-benzenediamine	C_12_H_12_N_2_	Cosmetic Ingredient	75 (Feces)
*N*,*N*-Dimethyl-*N*-benzyl-*N*-octadecylammonium	C_27_H_50_N^+^	Cosmetic Ingredient	100 (Feces)
*N*,*N*,*N*-Trimethyloctadecan-1-aminium	C_21_H_46_N^+^	Cosmetic Ingredient	100 (Feces)
Piperine	C_17_H_19_NO_3_	Food Additive	95 (Plasma) 85 (Feces)
Riboflavin	C_17_H_20_N_4_O_6_	Food Additive	100 (Urine) 85 (Feces)

a Classifications based on Teri et al.’s assignments, which were made using PubChem and the CompTox Chemicals Dashboard. For each compound, the detection frequency (DF) for each relevant matrix is listed. For detailed information regarding annotation thresholds and a complete list of identifications, see Tables S6 and S7, respectively.

Of these Level 1 identifications, 24 compounds were detected in feces, 18 in urine, and 7 in plasma. Several compounds were found exclusively in a single matrix, including lithocholic acid, isoxaben, and quaternary ammonium compounds in feces; dichlormid and deisopropylatrazine in urine; and deethylatrazine in plasma. The observed distribution of chemical features underscores the value of rapid, multimatrix profiling in human exposome studies. While feces contained the greatest number of unique compounds—consistent with its role as a repository of dietary, microbial, and xenobiotic metabolites^6,77,78^ —plasma and urine provided unique insight into the chemical exposome, capturing nonredundant exposures that were not detected in feces. Two *N*-dealkylated metabolites of atrazine, the widely applied chlorotriazine herbicide, were exclusively detected in plasma (deisopropylatrazine; DIA) and urine (deethylatrazine; DEA). Atrazine is rapidly and extensively metabolized to DIA, DEA, and downstream mercapturic acid derivatives in humans following oral and dermal absorption.^79^ Accordingly, urinary and plasma monitoring of DIA and DEA may provide a more reliable measure of human atrazine exposure while mitigating the risk of exposure underestimation; indeed, the parent compound was not detected in any matrix under our experimental conditions. Conversely, the detection of quaternary ammonium compounds (QACs) highlights the complementary insights provided by fecal profiling. Other groups and we have previously demonstrated that QACs—a large class of antimicrobial surfactants found in many cleaning, disinfecting, and personal care products—are not readily excreted into urine as parent compounds,^80–84^ but are typically recovered as a series of oxidized metabolites produced by the cytochrome P450 enzymes.^44,85^

Of the 8 ToxCast compounds detected in more than one matrix, 5 were broadly classified as “Food Additives”—reflecting their documented use, either naturally or synthetically, in food products. Caffeine and riboflavin (vitamin B2), for example, are frequently encountered in dietary sources (e.g., caffeine: coffee and tea; riboflavin: milk, eggs, and green vegetables),^86,87^ consistent with their relatively high detection frequencies (>65%). Piperine, a naturally occurring alkaloid present in black pepper^88^ and other dietary sources, similarly exhibited high detection frequencies in plasma (95%) and feces (85%). The artificial flavor additive ethylvanillin (3-ethoxy-4-hydroxybenzaldehyde) was also detected in urine, reflecting the highly diverse mixture of food-derived molecules in the human chemical exposome.

Tentative (Level 3) candidate structures are reported in cases where the available orthogonal evidence did not permit unambiguous assignment (Table S8). For example, multiple aromatic amines—the positional isomers 1- and 2-naphthylamine and methylquinolines (quinaldine, 6-methylquinoline, lepidine)—satisfied all annotation thresholds described in Table S6 but were not sufficiently resolved to select a single structure (Table S8). These compounds are widely recognized as persistent environmental pollutants due to their use in a variety of manufacturing processes (e.g., dyes, rubber, cosmetics, pharmaceuticals) and pesticide production.^71,89,90^ Many aromatic amines have been detected in cigarette smoke and exhibit carcinogenic activity; 2-naphthylamine, for example, is strongly associated with the occurrence of human bladder cancer^74,91^ and is currently classified as a Group 1 agent (“carcinogenic to humans”) by the International Agency for Research on Cancer (IARC).^92^ The methylquinoline isomers exhibit distinct toxicological profiles. In contrast to lepidine (4-methylquinoline), a potent hepatocarcinogen in mice and rats,^93,94^ quinaldine (2-methylquinoline) and 6-methylquinoline have shown minimal activity in initiation-promotion skin tumor bioassays^95^ and reduced genotoxicity in rat hepatocytes.^96^ Although these compounds were not sufficiently resolved by IM, LC, or MS/MS under our experimental conditions, the LC-IM-MS^E^ screen rapidly generated a high-quality list of targets to prioritize for confirmation and quantitation at higher confidence levels.

Internal concentrations measured in biological matrices are influenced by toxicokinetic processes, including absorption, distribution, metabolism, and excretion, which can substantially influence biomarker levels relative to external exposure concentrations.^97^ Because the present study focused on compound identification rather than quantitative exposure measurement, no *in vitro*-*in vivo* extrapolation (IVIVE) or toxicokinetic modeling was performed. However, future studies combining exposomics measurements with quantitative workflows could integrate internal concentration data with IVIVE or physiologically based pharmacokinetic (PBPK) models to estimate external exposures and support next-generation risk assessment.^98^ Biological factors (e.g., sex, age, BMI, and diet) can also influence matrix composition and background metabolite profiles, potentially leading to method performance variability across demographic groups.^99^ Although beyond the scope of the present study, systematic evaluations of workflow applicability to biologically diverse samples will be critical for population-level exposome studies.

As discussed above (see High-Throughput UPLC-IM-MS Analysis), detection biases stemming from the exclusive use of LC-ESI-MS are evident from our analysis and previous studies by Teri et al.^50^ Across both ESI+ and ESI– modes, 44.3% of all ToxCast chemical standards analyzed (2,075 of 4,684) were detected. The lowest detection frequencies were observed for chemical classes comprising nonpolar, poorly ionizable compounds, such as the hydrocarbons (saturated, unsaturated, polycyclic), vinyl halides, trialkylphosphites, benzodioxins, and thiophenic compounds, which lack functional groups capable of readily undergoing adduct formation during the electrospray process (see **Undetected Compounds** in the Supporting Information 2 for a complete list of detection frequencies per chemical class). Furthermore, as discussed by Teri et al., long-term chemical stability is a major factor contributing to detection and should be considered in future library development.

Our findings are generally consistent with those reported by Teri et al., who successfully measured the drift tube ^DT^CCS_N2_ values for 2,140 ToxCast chemical standards using ESI+, ESI–, and APCI+ coupled to LC-IM-HRMS, although there are a substantial number of unique CCS values present in either database. Notably, the authors detected 839 chemicals with APCI+ (39.2% of the 4,685 standards analyzed), but only 55 were uniquely detected with this source and polarity. The use of APCI enabled Teri et al. to determine ^DT^CCS_N2_ values for 9 polycyclic aromatic hydrocarbons and additional low- to mid-polarity chemical exposure agents not amenable to LC-ESI (e.g., azo dyes, pyrazine food additives, heterocyclic pesticides). In both databases, a substantial portion of the undetected standards are relatively nonpolar, volatile/semivolatile small molecules suitable for analysis by gas chromatography (GC)-MS (Figure S3). The benefits of GC-APCI-HRMS platforms for expanded analytical coverage in exposome analysis have recently been demonstrated by Izquierdo-Sandoval et al.^100^ The exclusive use of reversed-phase (RP)-LC is another limitation of the current study, and given the physicochemical diversity of potential small molecule exposures,^94^ future studies involving complementary techniques (e.g., hydrophilic interaction chromatography) present opportunities to further increase the detectable space of chemical exposomics.

Furthermore, while our triplicate retention time measurements were highly reproducible (∼89% of compounds displayed interday RSDs < 1%), the values reported in ToxBase should be interpreted as method-specific reference values due to the multitude of factors influencing LC separation (e.g., pump mixing behavior, dwell volume, temperature, and pressure fluctuations). Interlaboratory transferability could be further improved in the future with the incorporation of retention time indexing strategies. Although these systems, which typically rely on homologous series of reference compounds to normalize retention behavior across analytical platforms, are routinely employed in GC-MS, they have not yet been universally adopted in LC-MS due to the greater variety of detectable analytes.^102,103^ On the other hand, retention time calibration using several anchor analytes across the retention time range, as long as similar columns and gradients are used, is another approach to improve the transferability of chromatographic data across separation systems.^104^ To facilitate other users adopting the retention time calibration approach, we have identified a panel of commercially available ToxCast compounds spanning the ToxBase chromatographic range that could serve as anchor standards for retention time calibration (see **RT Calibration** in Supporting Information). Future work to validate this approach and harmonize retention data between different LC-HRMS systems will significantly enhance the utility of multidimensional reference databases.

Collectively, these results indicate that structurally diverse ToxCast compounds serving a variety of functions—including pesticides, cosmetic ingredients, and food additives—are detectable in humans. These compounds were confidently identified using ToxBase, a freely available public resource that currently includes multidimensional reference data for over 2,000 unique compounds. Notably, our database expands the analytical coverage of HRMS-based chemical exposomics by providing CCS values and retention times for 3,598 structurally diverse ions (2,346 cations and 1,252 anions), including >700 chemical exposure agents not reported in previous studies (Table S9). ToxBase also contains MS/MS fragmentation data for 1,629 unique compounds (∼79% of the database) in the common Mass Spectral Library (MSP) file format, providing researchers across laboratories and instrumental platforms a single, comprehensive tool for rapidly screening a wide range of chemical exposure agents in multidimensional HRMS datasets. Future studies incorporating quantitative analysis and targeted validation will be necessary to assess exposure levels of emerging contaminants and interindividual variability in populations of interest.

## Supplementary Material





## Notes

Source parameters and experimental conditions for UPLC-IM-MS and UPLC-MS/MS analysis, CCS calibration, QC summary and Skyline settings for human biofluid studies, adducts and superclasses in ToxBase, detected ToxCast compounds in human biofluids, evaluation of undetected compounds, and comparison between ToxBase and other reference databases (PDF)
All ESI+ CCS, ESI– CCS, and retention time values in ToxBase, compounds with multiple retention time and drift time peaks, chemical classifications of ToxCast database, comparison between ToxBase and literature TWIMCCSN2 values, detection frequencies per chemical class, retention time calibration standards, analytical performance, matrix effects, and recoveries (XLSX)

## ∥

Biological Sciences Division, Pacific Northwest National Laboratory, Richland, WA 99352
