# Supplementary material for: ToxBase: A Multidimensional ToxCast Reference Database for High-Throughput Human Exposome Analysis
Source: Environ Sci Technol. 2026 Jun 9;60(24):17314–27. doi: 10.1021/acs.est.5c18068 (PMC13296496; doi:10.1021/acs.est.5c18068)
Supplement: Supplementary file 1 [file es5c18068_si_001.pdf]

Supporting Information

ToxBase: A Multidimensional ToxCast Reference Database for High-Throughput Human Exposome Analysis

Ryan Nguyen[1], Griffin Rangel[1], Xingzhu Liu[1], Reuben A. Santoso[1], Amogh Bantwal[1], Dylan H. Ross[2], Ryan P. Seguin[1], Jennifer Liem[3], Yvonne S. Lin[3], Brian Pratt[4], Brendan X. MacLean[4], Michael J. MacCoss[4], Libin Xu*[,1]

[1]Department of Medicinal Chemistry, University of Washington, Seattle, WA 98195

[2]Biological Sciences Division, Pacific Northwest National Laboratory, Richland, WA 99352

[3]Department of Pharmaceutics, University of Washington, Seattle, WA 98195

[4]Department of Genome Sciences, University of Washington, Seattle, WA 98195

*Correspondence:

Libin Xu, Ph.D.

Tel: (206) 543-1080

Fax: (206) 685-3252

Email: libinxu@uw.edu

NO. OF PAGES: 19

NO. OF FIGURES: 3

NO. OF TABLES: 9

TABLE OF CONTENTS

**Table S1.** UPLC-IM-MS instrument parameters…………………………………………	SI-3
**Figure S1.** Representative CCS calibration curve………………………………………..	SI-4
**Table S2.** UPLC-MS/MS instrument conditions…………………................................	SI-5
**Table S3.** Example ToxBase library entry……………………………………..................	SI-6
**Table S4.** UPLC-IM-MS^{E} instrument conditions…………………………………………..	SI-7
**Table S5.** Quality control for human biofluid analysis……………………………………	SI-8
**Table S6.** Skyline analysis of human biofluid extracts…………………………………...	SI-9
**Figure S2.** Adducts and Superclasses in ToxBase…..…………………………………..	SI-10
**Table S7.** Level 1 detected ToxCast compounds………………………………………...	SI-11
**Table S8.** Level 3 detected ToxCast compounds………………………………………...	SI-15
**Figure S3.** Evaluation of undetected ToxCast compounds……………………………...	SI-16
**Table S9.** Comparison between IMFrag and other reference databases……………...	SI-17

Table S1. UPLC-IM-MS instrument parameters utilized for analysis of the ToxCast chemical reference standards.

Instrument Parameter	ESI+	ESI−
Source Temperature (°C)	130	130
Capillary Voltage (kV)	2.5	2
Cone Gas Flow (L/h)	90	90
Nebulizer Gas Flow (bar)	5	6.5
Desolvation Gas Flow (L/h)	800	800
Desolvation Temperature (°C)	500	500
Cone Voltage (V)	30	15
Source Offset (V)	10	10
IMS Wave Velocity (m/s)	500	Linear ramp 321 → 794
IMS Wave Height (V)	40	Linear ramp 22 → 40

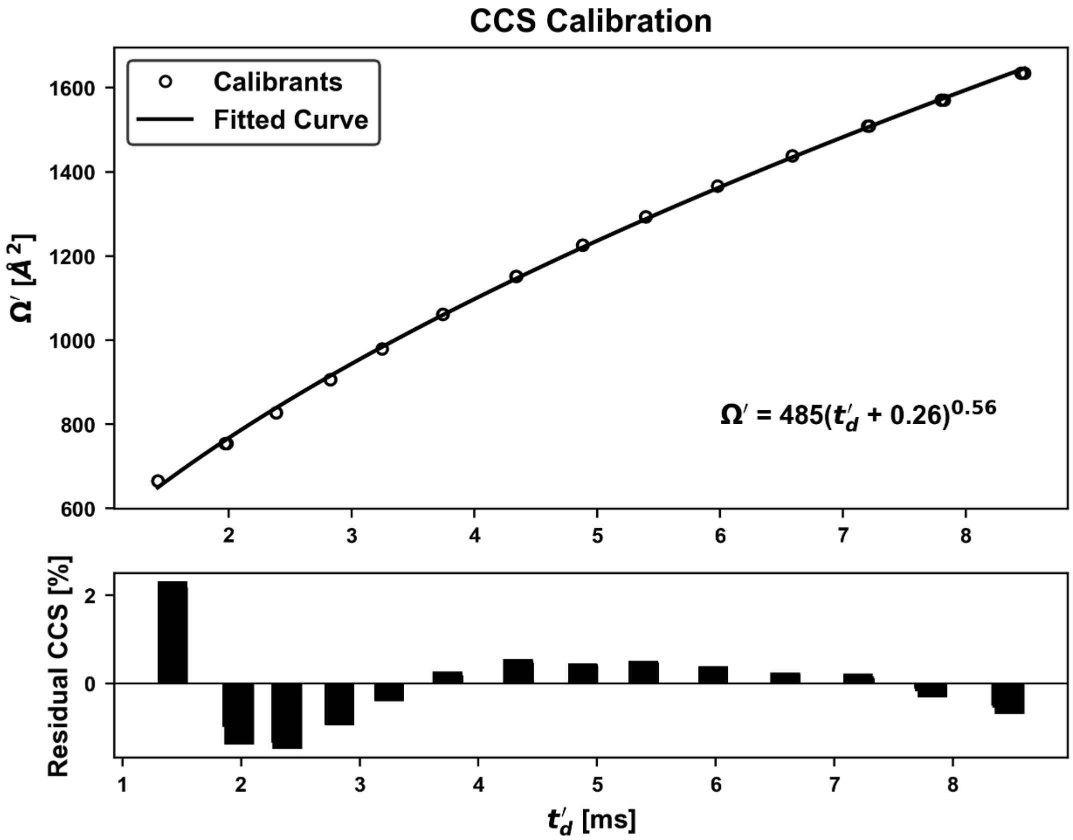

Figure S1. Representative CCS calibration plot for PolyAla displaying the relationship between corrected drift time (t_d') and correct CCS (Ω'). The lower panel displays the residual error between experimental and fitted CCS values, expressed as percent CCS deviation.

Table S2. UPLC-MS/MS data-dependent analysis (DDA) instrument conditions utilized for assembly of the ToxBase MS/MS reference library.

Parameter	Value
Survey Start Mass	50
Survey End Mass	1200
MS/MS Switch Threshold	Intensity > 10000
Number of Precursors	5
Survey Scan Time (s)	0.20
Survey Interscan Time (s)	0.01
Exclude Detected Precursor (s)	5
MS/MS Start Mass	50
MS/MS End Mass	1200
MS/MS Switchback Threshold	TIC > 100000
MS/MS Scan Time (s)	0.10
MS/MS Interscan Time (s)	0.01
Transfer MS/MS Collision Energy Ramp Low Mass Start (eV)	5
Transfer MS/MS Collision Energy Ramp Low Mass End (eV)	40
Transfer MS/MS Collision Energy Ramp High Mass Start (eV)	20
Transfer MS/MS Collision Energy Ramp High Mass Start (eV)	70

Table S3. Example ToxBase library entry. The provided library follows the simple NIST Text file format[1], wherein each spectrum begins with the required “NAME” (i.e., compound name) Field title. Additional Fields in ToxBase, which are not explicitly required, include: “PRECURSORMZ” (precursor theoretical *m/z*), “PRECURSORTYPE” (adduct), “IONMODE” (ionization mode), “FORMULA” (chemical formula), “RETENTIONTIMEMINS” (measured retention time in min), and “CCS_SQA” (measured CCS value in Å^2). The “NUM PEAKS” Field is required and defines the number of entries in the *m/z* and intensity list. Each Field title must be on a separate line, but the peaks in the provided mass spectrum are not required to be ordered or normalized.

NAME:	Caffeine
PRECURSORMZ:	195.0877
PRECURSORTYPE:	[M + H]+
IONMODE:	Positive
FORMULA:	C8H10N4O2
RETENTIONTIMEMINS:	0.81
CCS_SQA:	137.37
NUM PEAKS:	207
…	…
133.0844	999
151.0947	259
…	…

Table S4. UPLC-IM-MS^E instrument conditions utilized for the analysis of human biofluid extracts.

Parameter	Value
Low Energy Start Mass	50
Low Energy End Mass	1200
Low Energy Scan Time (s)	0.500
Low Energy Interscan Time (s)	0.014
High Energy Start Mass	50
High Energy End Mass	1200
High Energy Scan Time (s)	0.500
High Energy Interscan Time (s)	0.014
Transfer Collision Energy Ramp Start (eV)	25
Transfer Collision Energy Ramp End (eV)	45

Table S5. Analytical performance of deuterated benzalkonium chloride (d_7-BAC) internal standards across human biofluids. Internal standards were spiked into each biofluid sample prior to extraction at 10 pmol (feces) 2.5 pmol (urine), and 0.3 pmol (plasma) to assess chromatographic stability, mass accuracy, and signal repeatability throughout the analytical sequence.

**Matrix**[a]	**Internal Standard**[b]	**RT (min) (mean ± SD)**	**Mass Error (ppm) (mean ± SD)**	**Mean MS^1 Peak Area**	**MS^1 Peak Area RSD (%)**
Feces	d_7-C_{10}-BAC	0.850 ± 0.000	1.45 ± 1.64	14,885	30.7
Feces	d_7-C_{12}-BAC	0.880 ± 0.000	1.07 ± 1.06	42,740	32.5
Feces	d_7-C_{14}-BAC	0.912 ± 0.006	0.73 ± 1.17	77,028	28.9
Feces	d_7-C_{16}-BAC	0.970 ± 0.000	1.05 ± 0.88	110,174	27.0
Urine	d_7-C_{10}-BAC	0.850 ± 0.000	1.38 ± 1.23	17,391	62.6
Urine	d_7-C_{12}-BAC	0.880 ± 0.000	0.59 ± 1.15	40,851	51.2
Urine	d_7-C_{14}-BAC	0.914 ± 0.008	0.66 ± 0.95	77,603	46.5
Urine	d_7-C_{16}-BAC	0.970 ± 0.000	1.15 ± 0.75	149,470	40.2
Plasma	d_7-C_{10}-BAC	0.850 ± 0.000	0.92 ± 1.27	24,733	25.9
Plasma	d_7-C_{12}-BAC	0.880 ± 0.000	0.42 ± 0.68	42,935	28.4
Plasma	d_7-C_{14}-BAC	0.910 ± 0.000	0.65 ± 1.64	45,818	33.5
Plasma	d_7-C_{16}-BAC	0.970 ± 0.000	0.92 ± 1.32	42,666	45.4

[a] n = 20 for each matrix.

[b] Precursor *m/z* values for the $[M]^+$ adduct of d_7-C_{10}-BAC, d_7-C_{12}-BAC, d_7-C_{14}-BAC, and d_7-C_{16}-BAC are: 283.3125, 311.3438, 339.3751, and 367.4064, respectively.

Table S6. Description of confidence levels[2] and annotation thresholds utilized in the Skyline analysis of human biofluids. Prior to manually inspecting features of interest in Skyline, the complete list of extracted peaks – for quality controls, procedural blanks, and biofluid samples – was exported as a .csv file using the Document Grid function. Following quality control procedures (see **Table S4)**, precursor signal intensities observed in study samples were compared against those detected in matrix-matched procedural blanks for each compound. Features displaying an MS1 area less than three times the standard deviation[3] of the target analyte in procedural blanks were removed from the feature list and not further processed. The filtered data were then manually reviewed in Skyline, primarily to assess MS1 peak quality and to confirm the presence of mobility-aligned fragments when possible. Following these steps, the features were carefully assigned a confidence level using the definitions[4] and annotation thresholds defined below.

Confidence Level	Definition	Annotation Thresholds[a]
1	**Confirmed Structure:** Single structure verified by analysis of an authentic reference standard under identical conditions on the same instrument. Level 1 identifications meet all the (1) exact m/z, (2) isotopic pattern, (3) CCS, and (4) RT Annotation Thresholds. Evidence of mobility-aligned fragments matching a reference spectrum should also be confirmed or investigated, when possible.	**Exact *m/z*:** Measured *m/z* is ± 5 ppm of the exact *m/z*.<br>**Isotopic Pattern:** Visually matches theoretical formula distribution; "idotp" ≥ 0.7.<br>**CCS:** Fixed IM filtering (0.3 ms) centered on the reference DT.<br>**RT:** Measured RT is ± 0.1 min of the reference RT.<br>**MS/MS:** Visual confirmation of mobility-aligned reference fragments, when possible; "dotp" ≥ 0.5.
3	**Tentative Candidate Structures:** At least one candidate structure consistent with available experimental evidence. Level 3 identifications meet all the (1) exact m/z, (2) isotopic pattern, (3) CCS, and (4) RT Annotation Thresholds, but more than one candidate structure may be proposed. When possible, mobility-aligned fragmentation data may be utilized to promote a "most likely" candidate or define "tentative" candidates.	**Exact *m/z*:** Measured *m/z* is ± 5 ppm of the exact *m/z*.<br>**Isotopic Pattern:** Visually matches theoretical formula distribution; "idotp" ≥ 0.7.<br>**CCS:** Fixed IM filtering (0.3 ms) centered on the reference DT.<br>**RT:** Measured RT is ± 0.1 min of the reference or predicted RT.<br>**MS/MS:** Visual confirmation of mobility-aligned reference or predicted fragments, when possible; "dotp" ≥ 0.5.

[a]Reference values for Level 1 identifications are *strictly limited* to data from authentic standards analyzed under identical conditions on the same instrument. In contrast, Level 3 identifications may be proposed using library (in-house) *or* in-silico predicted values. All reported Level 1 and Level 3 identifications herein are based on in-house ToxBase reference data.

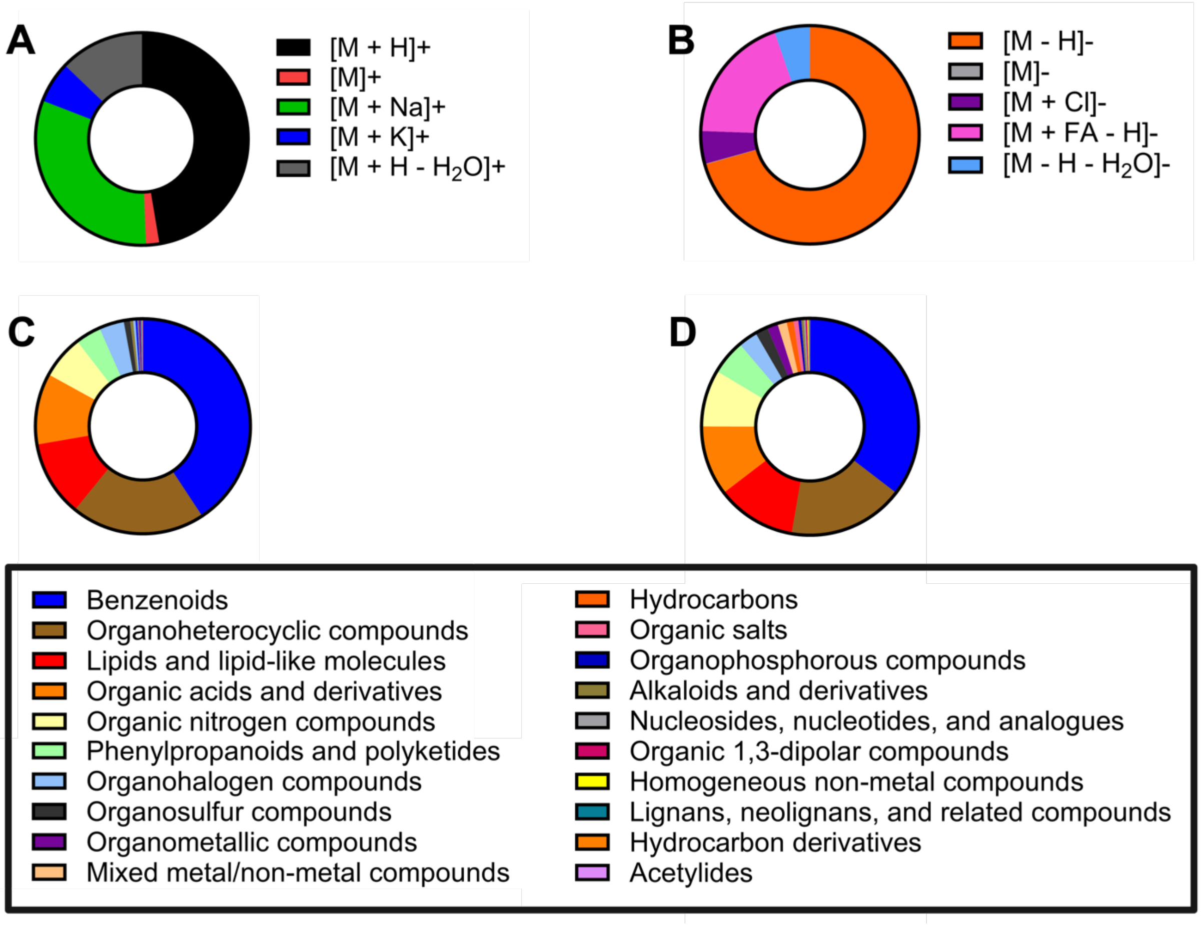

Figure S2. Distribution of (A) ESI+ and (B) ESI− adducts represented in the ToxBase database. Distribution of superclasses[5] represented in (C) ToxBase and (D) the ToxCast library.

Table S7. Complete list of ToxCast compounds detected in human biofluids identified with Level 1 confidence. Detection frequencies (DF) are provided for each relevant matrix.

Candidate Structure	Formula	Classification[a]	DF (%) (Matrix)
(9Z,12R)-12-Hydroxyoctadec-9-enoic acid	$C_{18}H_{34}O_3$	Cosmetic Ingredient	65 (Feces)
1,2-Dimethyl-5-nitroimidazole	$C_5H_7N_3O_2$	Pesticide	85 (Plasma)
1,3-Diiminobenz(f)isoindoline	$C_{12}H_9N_3$	Chemical Industrial	10 (Plasma)
12-Hydroxyoctadecanoic acid	$C_{18}H_{36}O_3$	Chemical Industrial	70 (Feces)
2-Allyloxymethyl-2-ethylpropanediol	$C_9H_{18}O_3$	Chemical Industrial	35 (Feces)
2-Phenylbenzimidazole	$C_{13}H_{10}N_2$	Chemical Industrial	20 (Urine)
2'-Aminoacetophenone	C_8H_9NO	Food Additive	90 (Feces)
3-[Ethyl(phenyl)amino]propanenitrile	$C_{11}N_{14}N_2$	Chemical Industrial	45 (Feces)
3-Ethoxy-4-hydroxybenzaldehyde	$C_9H_{10}O_3$	Food Additive	35 (Urine)
4-Anilinophenol	$C_{12}H_{11}NO$	Pesticide	100 (Urine)<br>85 (Feces)
4-Dimethylaminobenzaldehyde	$C_9H_{11}NO$	Chemical Industrial	95 (Feces)
4-Nitrosodiphenylamine	$C_{12}H_{10}N_2O$	Chemical Industrial	85 (Urine)

[a]Classifications based on Teri et al.[2] assignments, which were made using PubChem and the CompTox Chemicals Dashboard.

Candidate Structure	Formula	Classification	DF (%) (Matrix)
5-Ethyl-2,3-pyridinedicarboxylic acid	$C_9H_9NO_4$	Chemical Industrial	100 (Urine)
8-Hydroxyquinoline	C_9H_7NO	Cosmetic Ingredient	95 (Plasma)<br>95 (Urine)<br>100 (Feces)
Ancymidol	$C_{15}H_{16}N_2O_2$	Pesticide	85 (Feces)
Apigenin	$C_{15}H_{10}O_5$	Food Additive	30 (Urine)<br>35 (Feces)
Benzalkonium chloride C14	$C_{23}H_{42}N+$	Cosmetic Ingredient	100 (Feces)
Benzo(f)quinoline	$C_{13}H_9N$	Polycyclic Aromatic Hydrocarbons	85 (Urine)
Benzyldimethyldodecylammonium	$C_{17}H_{30}N+$	Cosmetic Ingredient	65 (Feces)
Benzylhexadecyldimethylammonium	$C_{25}H_{46}N+$	Cosmetic Ingredient	95 (Feces)
Caffeine	$C_8H_{10}N_4O_2$	Food Additive	55 (Plasma)<br>65 (Urine)
Cetylpyridinium	$C_{21}H_{38}N+$	Pharma-ceutical	45 (Feces)
Decamethylcyclopentasiloxane	$C_{10}H_{30}O_5Si_5$	Cosmetic Ingredient	100 (Urine)
Deethylatrazine	$C_6H_{10}ClN_5$	Pesticide	75 (Plasma)
Deisopropylatrazine	$C_5H_8ClN_5$	Pesticide	75 (Urine)

Candidate Structure	Formula	Classification	DF (%) (Matrix)
Dichlormid	$C_8H_{11}Cl_2NO$	Pesticide	50 (Urine)
Didecyldimethylammonium chloride	$C_{22}H_{48}N+$	Cosmetic Ingredient	85 (Feces)
Diketometribuzin	$C_7H_{12}N_4O_2$	Pesticide	70 (Urine)
Dimethyl 3,3'-thiodipropionate	$C_8H_{14}O_4S$	Chemical Industrial	55 (Urine)
Ethylhexadecyldimethylammonium	$C_{20}H_{44}N+$	Cosmetic Ingredient	95 (Urine)<br>80 (Feces)
Famphur	$C_{10}H_{16}NO_5PS_2$	Pesticide	20 (Urine)
Isoxaben	$C_{18}H_{25}N_2O_4$	Pesticide	35 (Feces)
Lithocholic acid	$C_{24}H_{40}O_3$	Bile Acids and Derivatives	90 (Feces)
N-Butylbenzenesulfonamide	$C_{10}H_{15}NO_2S$	Chemical Industrial	50 (Feces)
N-Phenyl-1,4-benzenediamine	$C_{12}H_{12}N_2$	Cosmetic Ingredient	75 (Feces)
N,N-Dimethyl-N-benzyl-N-octadecylammonium	$C_{27}H_{50}N+$	Cosmetic Ingredient	100 (Feces)
N,N,N-Trimethyloctadecan-1-aminium	$C_{21}H_{46}N+$	Cosmetic Ingredient	100 (Feces)

Candidate Structure	Formula	Classification	DF (%) (Matrix)
Piperine	$C_{17}H_{19}NO_3$	Food Additive	95 (Plasma)<br>85 (Feces)
Riboflavin	$C_{17}H_{20}N_4O_6$	Food Additive	100 (Urine)<br>85 (Feces)
Thiabendazole	$C_{10}H_7N_3S$	Pesticide	15 (Urine)
Thiofanox	$C_9H_{18}N_2O_2S$	Pesticide	10 (Plasma)

Table S8. Complete list of ToxCast compounds detected in human biofluids identified with Level 3 confidence. All possible candidate structures are reported, per the current guidance for IM-MS data[4].

Nr.	Candidate Structures	Formula	Matrices
1.	Quinoline<br>Isoquinoline	C_9H_7N	Plasma, Urine, Feces
2.	1-Naphthylamine<br>2-Naphthylamine<br>Quinaldine<br>6-Methylquinoline<br>Lepidine<br>[a]6-Ethenyl-1H-indole (*in-source fragment of Tryptophan)*	$C_{10}H_9N$	Plasma, Urine, Feces
3.	1,7-Dimethylxanthine<br>Theophylline<br>Theobromine	$C_7H_8N_4O_2$	Plasma, Urine, Feces

[a]These candidate isomers share an exact mass and several MS/MS fragments with 6-Ethenyl-1H-indole, a known *in-source fragment* of tryptophan[6]. As such, tryptophan is also considered a candidate structure despite not being represented in either the ToxCast chemical library or the current ToxBase database.

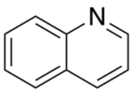

Quinoline

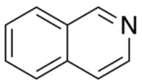

Isoquinoline

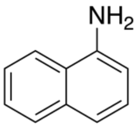

1-Naphthylamine

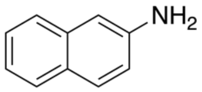

2-Naphthylamine

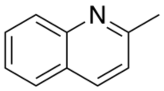

Quinaldine

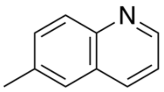

6-Methylquinoline

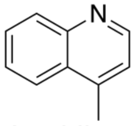

Lepidine

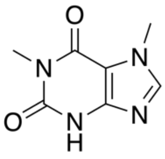

1,7-Dimethylxanthine

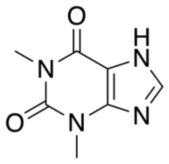

Theophylline

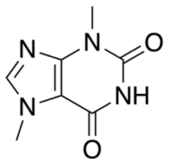

Theobromine

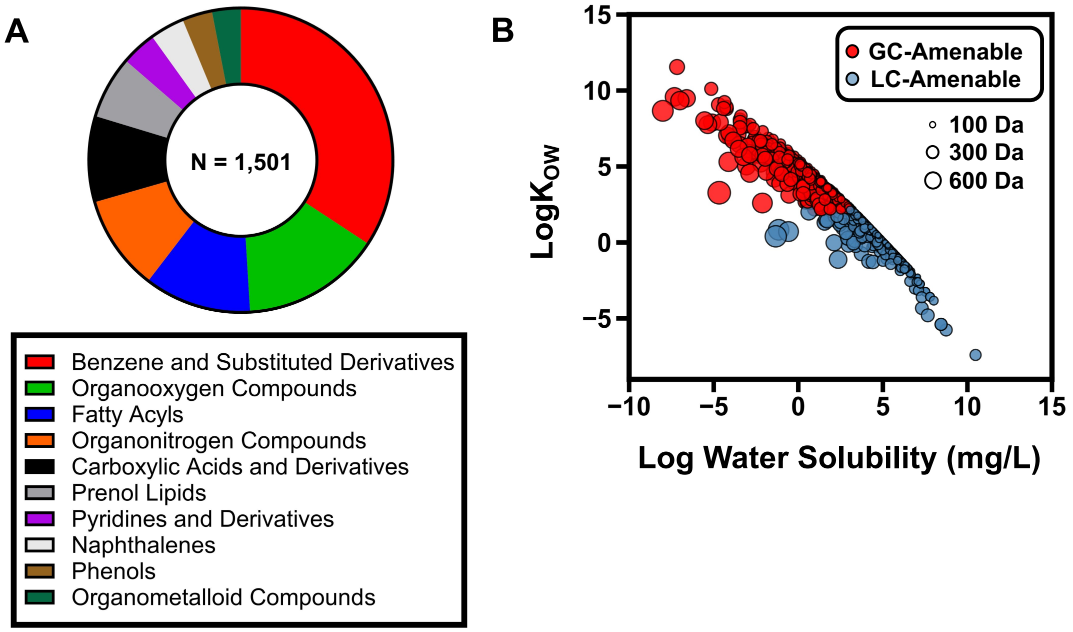

Figure S3. (A) Pi chart depicting the chemical classes of 1,501 compounds that were not detected by LC-ESI(+/-)-IM-MS analysis. Only the top 10 classes by abundance are shown – for a complete list of detection frequencies per chemical class, see the **Supporting Information** (Excel spreadsheet, "Undetected Compounds" tab). (B) Chemical space of the undetected ToxCast compounds. Relatively non-polar, volatile/semi-volatile GC-amenable analytes with a logarithm of water solubility in mg/L ≤ 3 and logarithm of the octanol-water partition coefficient $K_{OW} \geq 2$ are depicted in red[7]. Non-volatile analytes displaying moderate or high aqueous solubility that are typically suitable for LC-based methods are colored in blue. Water solubility and K_{OW} values were estimated using WSKOWWIN-style regression[8] and RDKit's Wildman-Crippen LogP function[9], respectively.

Table S9. Comparison between ToxBase and other reference databases relevant to exposomics.

Resource	Chemical Domain	CCS	RT/RI[a]	MS/MS	Data Formats
ToxBase	Environmental (ToxCast)	$^{TWIM}CCS_{N2}$	Yes	Yes	MSP, XLSX, Web
Teri et al.[2]	Environmental (ToxCast)	$^{DT}CCS_{N2}$	No	No	XLSX
METLIN-CCS[10]	Endogenous, Environmental	$^{TIMS}CCS_{N2}$[b]	No[11]	No[12]	CSV
HMDB[13]	Endogenous, Environmental	Yes	Yes	Yes	XML, TXT, PNG, mzML, CSV, JSON, Web
PubChemLite[14]	Endogenous, Environmental	Yes	No	No	CSV, Web
MoNA[15]	Endogenous, Environmental	No	No	Yes	JSON, MSP, Web
GNPS[16]	Endogenous, Environmental	No	No	Yes	MGF, MSP, JSON, Web
Song et al.[17]	Environmental (Food Contact Materials)	$^{TWIM}CCS_{N2}$	No	No	XLSX

[a]Retention index (RI). Note that "Yes" indicates the feature (i.e., CCS, RT/RI, MS/MS) is provided for at least a subset of the compounds in the resource.

[b]Trapped ion mobility spectrometry (TIMS). The METLIN-CCS database provides CCS values for over 27,500 molecular standards. METLIN contains MS/MS data at multiple energies for nearly one million molecular standards at multiple energies, including endogenous small molecules (e.g., lipids, peptides, carbohydrates) and environmental toxicants. Experimental retention time information for approximately 80,000 METLIN molecular standards was assembled by Domingo-Almenara et al. as part of the METLIN small molecule retention time (SMRT) dataset.

References

(1) Stein, S. E.; Mikaia, A.; Linstrom, P.; Mirokhin, Y.; Tchekhovskoi, D.; Yang, X.; Mallard, W. G.; Sparkman, O. D.; Sparkman, J. A. For Use with Microsoft® Windows User's Guide.

(2) Teri, D.; Aly, N. A.; Dodds, J. N.; Zhang, J.; Thiessen, P. A.; Bolton, E. E.; Joseph, K. M.; Williams, A. J.; Schymanski, E. L.; Rusyn, I.; Baker, E. S. Reference Library for Suspect Screening of Environmental Toxicants Using Ion Mobility Spectrometry-Mass Spectrometry. *Commun Chem* **2025**, *8* (1), 224. https://doi.org/10.1038/s42004-025-01619-7.

(3) Zheng, G.; Webster, T. F.; Salamova, A. Quaternary Ammonium Compounds: Bioaccumulation Potentials in Humans and Levels in Blood before and during the Covid-19 Pandemic. *Environmental Science & Technology* **2021**, *55* (21), 14689–14698. https://doi.org/10.1021/acs.est.1c01654.

(4) Boatman, A. K.; Chappel, J. R.; Kirkwood-Donelson, K. I.; Fleming, J. F.; Reif, D. M.; Schymanski, E. L.; Rager, J. E.; Baker, E. S. Updated Guidance for Communicating PFAS Identification Confidence with Ion Mobility Spectrometry. *Environ. Sci. Technol.* **2025**, *59* (33), 17711–17721. https://doi.org/10.1021/acs.est.5c01354.

(5) Djoumbou Feunang, Y.; Eisner, R.; Knox, C.; Chepelev, L.; Hastings, J.; Owen, G.; Fahy, E.; Steinbeck, C.; Subramanian, S.; Bolton, E.; Greiner, R.; Wishart, D. S. ClassyFire: Automated Chemical Classification with a Comprehensive, Computable Taxonomy. *Journal of Cheminformatics* **2016**, *8* (1), 61. https://doi.org/10.1186/s13321-016-0174-y.

(6) Asakawa, D.; Mizuno, H.; Sugiyama, E.; Todoroki, K. Fragmentation Study of Tryptophan-Derived Metabolites Induced by Electrospray Ionization Mass Spectrometry for Highly Sensitive Analysis. *Analyst* **2021**, *146* (7), 2292–2300. https://doi.org/10.1039/D0AN02069A.

(7) US EPA, O. *Technical Overview of Volatile Organic Compounds*. https://www.epa.gov/indoor-air-quality-iaq/technical-overview-volatile-organic-compounds (accessed 2025-10-16).

(8) *Improved method for estimating water solubility from octanol/water partition coefficient - Meylan - 1996 - Environmental Toxicology and Chemistry - Wiley Online Library*. https://onlinelibrary.wiley.com/doi/abs/10.1002/etc.5620150205 (accessed 2026-03-04).

(9) *rdkit.Chem.Crippen module — The RDKit 2025.09.6 documentation*. https://www.rdkit.org/docs/source/rdkit.Chem.Crippen.html (accessed 2026-03-04).

(10) Baker, E. S.; Hoang, C.; Uritboonthai, W.; Heyman, H. M.; Pratt, B.; MacCoss, M.; MacLean, B.; Plumb, R.; Aisporna, A.; Siuzdak, G. METLIN-CCS: An Ion Mobility Spectrometry Collision Cross Section Database. *Nat Methods* **2023**, *20* (12), 1836–1837. https://doi.org/10.1038/s41592-023-02078-5.

(11) Domingo-Almenara, X.; Guijas, C.; Billings, E.; Montenegro-Burke, J. R.; Uritboonthai, W.; Aisporna, A. E.; Chen, E.; Benton, H. P.; Siuzdak, G. The METLIN Small Molecule Dataset for Machine Learning-Based Retention Time Prediction. *Nat Commun* **2019**, *10*, 5811. https://doi.org/10.1038/s41467-019-13680-7.

(12) Montenegro-Burke, J. R.; Guijas, C.; Siuzdak, G. METLIN: A Tandem Mass Spectral Library of Standards. *Methods Mol Biol* **2020**, *2104*, 149–163. https://doi.org/10.1007/978-1-0716-0239-3_9.

(13) Wishart, D. S.; Guo, A.; Oler, E.; Wang, F.; Anjum, A.; Peters, H.; Dizon, R.; Sayeeda, Z.; Tian, S.; Lee, B. L.; Berjanskii, M.; Mah, R.; Yamamoto, M.; Jovel, J.; Torres-Calzada, C.; Hiebert-Giesbrecht, M.; Lui, V. W.; Varshavi, D.; Varshavi, D.; Allen, D.; Arndt, D.; Khetarpal, N.; Sivakumaran, A.; Harford, K.; Sanford, S.; Yee, K.; Cao, X.; Budinski, Z.; Liigand, J.; Zhang, L.; Zheng, J.; Mandal, R.; Karu, N.; Dambrova, M.; Schiöth, H. B.; Greiner, R.; Gautam, V. HMDB 5.0: The Human Metabolome Database for 2022. *Nucleic Acids Res* **2022**, *50* (D1), D622–D631. https://doi.org/10.1093/nar/gkab1062.

(14) Elapavalore, A.; Ross, D. H.; Grouès, V.; Aurich, D.; Krinsky, A. M.; Kim, S.; Thiessen, P. A.; Zhang, J.; Dodds, J. N.; Baker, E. S.; Bolton, E. E.; Xu, L.; Schymanski, E. L. PubChemLite Plus Collision Cross Section (CCS) Values for Enhanced Interpretation of Nontarget Environmental Data. *Environ. Sci. Technol. Lett.* **2025**, *12* (2), 166–174. https://doi.org/10.1021/acs.estlett.4c01003.

(15) Neumann, S.; Meier, R.; Wenk, M.; Elapavalore, A.; Nishioka, T.; Schulze, T.; Stravs, M.; Tsugawa, H.; Matsuda, F.; Schymanski, E. L. MassBank: An Open and FAIR Mass Spectral Data Resource. *Nucleic Acids Res* **2026**, *54* (D1), D601–D606. https://doi.org/10.1093/nar/gkaf1193.

(16) Wang, M.; Carver, J. J.; Phelan, V. V.; Sanchez, L. M.; Garg, N.; Peng, Y.; Nguyen, D. D.; Watrous, J.; Kapono, C. A.; Luzzatto-Knaan, T.; Porto, C.; Bouslimani, A.; Melnik, A. V.; Meehan, M. J.; Liu, W.-T.; Crüsemann, M.; Boudreau, P. D.; Esquenazi, E.; Sandoval-Calderón, M.; Kersten, R. D.; Pace, L. A.; Quinn, R. A.; Duncan, K. R.; Hsu, C.-C.; Floros, D. J.; Gavilan, R. G.; Kleigrewe, K.; Northen, T.; Dutton, R. J.; Parrot, D.; Carlson, E. E.; Aigle, B.; Michelsen, C. F.; Jelsbak, L.; Sohlenkamp, C.; Pevzner, P.; Edlund, A.; McLean, J.; Piel, J.; Murphy, B. T.; Gerwick, L.; Liaw, C.-C.; Yang, Y.-L.; Humpf, H.-U.; Maansson, M.; Keyzers, R. A.; Sims, A. C.; Johnson, A. R.; Sidebottom, A. M.; Sedio, B. E.; Klitgaard, A.; Larson, C. B.; Boya P, C. A.; Torres-Mendoza, D.; Gonzalez, D. J.; Silva, D. B.; Marques, L. M.; Demarque, D. P.; Pociute, E.; O'Neill, E. C.; Briand, E.; Helfrich, E. J. N.; Granatosky, E. A.; Glukhov, E.; Ryffel, F.; Houson, H.; Mohimani, H.; Kharbush, J. J.; Zeng, Y.; Vorholt, J. A.; Kurita, K. L.; Charusanti, P.; McPhail, K. L.; Nielsen, K. F.; Vuong, L.; Elfeki, M.; Traxler, M. F.; Engene, N.; Koyama, N.; Vining, O. B.; Baric, R.; Silva, R. R.; Mascuch, S. J.; Tomasi, S.; Jenkins, S.; Macherla, V.; Hoffman, T.; Agarwal, V.; Williams, P. G.; Dai, J.; Neupane, R.; Gurr, J.; Rodríguez, A. M. C.; Lamsa, A.; Zhang, C.; Dorrestein, K.; Duggan, B. M.; Almaliti, J.; Allard, P.-M.; Phapale, P.; Nothias, L.-F.; Alexandrov, T.; Litaudon, M.; Wolfender, J.-L.; Kyle, J. E.; Metz, T. O.; Peryea, T.; Nguyen, D.-T.; VanLeer, D.; Shinn, P.; Jadhav, A.; Müller, R.; Waters, K. M.; Shi, W.; Liu, X.; Zhang, L.; Knight, R.; Jensen, P. R.; Palsson, B. Ø.; Pogliano, K.; Linington, R. G.; Gutiérrez, M.; Lopes, N. P.; Gerwick, W. H.; Moore, B. S.; Dorrestein, P. C.; Bandeira, N. Sharing and Community Curation of Mass Spectrometry Data with Global Natural Products Social Molecular Networking. *Nat Biotechnol* **2016**, *34* (8), 828–837. https://doi.org/10.1038/nbt.3597.

(17) Song, X.-C.; Canellas, E.; Dreolin, N.; Goshawk, J.; Nerin, C. A Collision Cross Section Database for Extractables and Leachables from Food Contact Materials. *J. Agric. Food Chem.* **2022**, *70* (14), 4457–4466. https://doi.org/10.1021/acs.jafc.2c00724.
